# Genetic variants affecting cross-sectional lung function in adults show little or no effect on longitudinal lung function decline

**DOI:** 10.1136/thoraxjnl-2016-208448

**Published:** 2017-02-07

**Authors:** Catherine John, María Soler Artigas, Jennie Hui, Sune Fallgaard Nielsen, Nicholas Rafaels, Peter D Paré, Nadia N Hansel, Nick Shrine, Iain Kilty, Anders Malarstig, Scott A Jelinsky, Signe Vedel-Krogh, Kathleen Barnes, Ian P Hall, John Beilby, Arthur W Musk, Børge G Nordestgaard, Alan James, Louise V Wain, Martin D Tobin

**Affiliations:** 1 Genetic Epidemiology Group, Department of Health Sciences, University of Leicester, Leicester, UK; 2 School of Pathology and Laboratory Medicine, The University of Western Australia, Australia; 3 PathWest, Department of Health, Government of Western Australia, Perth, WA, Australia; 4 Busselton Population Medical Research Institute, Sir Charles Gairdner Hospital, Western Australia, Australia; 5 School of Population Health, The University of Western Australia, Australia; 6 Department of Clinical Biochemistry, Herlev and Gentofte Hospital, Copenhagen University Hospital, Denmark; 7 Center for Personalized Medicine and Biomedical Informatics, School of Medicine, University of Colorado, Anschutz Medical Campus; 8 University of British Columbia Centre for Heart Lung Innovation, St Paul's Hospital, Vancouver, British Columbia, Canada; 9 Department of Medicine, School of Medicine, Johns Hopkins University, Baltimore, Maryland, USA; 10 Pfizer Worldwide Research and Development, Cambridge, Massachusetts, USA; 11 Pfizer Worldwide Research and Development, Stockholm, Sweden; 12 Division of Respiratory Medicine, Queen's Medical Centre, University of Nottingham, Nottingham, UK; 13 Department of Respiratory Medicine, Sir Charles Gairdner Hospital, Nedlands, Western Australia, Australia; 14 School of Medicine and Pharmacology, The University of Western Australia, Australia; 15 Department of Pulmonary Physiology and Sleep Medicine/West Australian Sleep Disorders Research Institute, Sir Charles Gairdner Hospital, Nedlands, Western Australia, Australia; 16 National Institute for Health Research (NIHR) Leicester Respiratory Biomedical Research Unit, Glenfield Hospital, Leicester, UK

**Keywords:** COPD epidemiology, COPD ÀÜ Mechanisms, Lung Physiology, Respiratory Measurement

## Abstract

**Background:**

Genome-wide association studies have identified numerous genetic regions that influence cross-sectional lung function. Longitudinal decline in lung function also includes a heritable component but the genetic determinants have yet to be defined.

**Objectives:**

We aimed to determine whether regions associated with cross-sectional lung function were also associated with longitudinal decline and to seek novel variants which influence decline.

**Methods:**

We analysed genome-wide data from 4167 individuals from the Busselton Health Study cohort, who had undergone spirometry (12 695 observations across eight time points). A mixed model was fitted and weighted risk scores were calculated for the joint effect of 26 known regions on baseline and longitudinal changes in FEV_1_ and FEV_1_/FVC. Potential additional regions of interest were identified and followed up in two independent cohorts.

**Results:**

The 26 regions previously associated with cross-sectional lung function jointly showed a strong effect on baseline lung function (p=4.44×10^−16^ for FEV_1_/FVC) but no effect on longitudinal decline (p=0.160 for FEV_1_/FVC). This was replicated in an independent cohort. 39 additional regions of interest (48 variants) were identified; these associations were not replicated in two further cohorts.

**Conclusions:**

Previously identified genetic variants jointly have a strong effect on cross-sectional lung function in adults but little or no effect on the rate of decline of lung function. It is possible that they influence COPD risk through lung development. Although no genetic variants have yet been associated with lung function decline at stringent genome-wide significance, longitudinal change in lung function is heritable suggesting that there is scope for future discoveries.

**Key messages**

**What is the key question?**

- Do genetic regions associated with cross-sectional lung function also show association with rate of decline?

**What is the bottom line?**

- Twenty-six regions associated with cross-sectional lung function in adults—many of them associated with COPD risk—were not associated with rate of decline in lung function.

**Why read on?**

- These findings provide an important insight into the possible pathways through which known genetic regions influence lung function and risk of COPD.

## Introduction

Reduction of FEV_1_ relative to FVC defines COPD, one of the leading causes of death worldwide. Measures of lung function are also important predictors of morbidity and mortality in the general population.^1–3^ While environmental factors, particularly smoking, impact lung function, genetic variation is also a major determinant.^4^

Genome-wide association studies (GWAS) to date have identified numerous regions associated with lung function measured at a single point in time (ie, cross-sectional lung function).^5–9^ The lung function attained at a given time point in adulthood will be influenced by factors that affect either the development of lung function in earlier life or the rate of subsequent decline in lung function or both. Both cross-sectional lung function and longitudinal change in lung function are heritable. Although heritability estimates for longitudinal decline in lung function range between 10% and 39%,^10^ the individual genetic determinants have yet to be defined. Identifying the responsible genes could provide a promising route for intervention in COPD, since this is typically diagnosed well after lung function has reached its peak and modifying its further decline could prove to be a feasible therapeutic option.

The objectives of our study were as follows: (1) to examine the association with longitudinal change for those regions previously identified as significantly associated with cross-sectional FEV_1_ or FEV_1_/FVC and (2) to seek novel variants which reach genome-wide significance for association with longitudinal change in lung function, using a cohort with multiple lung function measurements over an extended period of up to 40 years and an imputation panel which provides dense coverage of both common and low-frequency variants.

## Methods

### Discovery data source and study population

The Busselton Health Study (BHS) is a longitudinal health survey that began in 1966 in the town of Busselton in the southwestern region of Western Australia. In 1994/1995, a cross-sectional community follow-up study was undertaken where blood was taken for DNA extraction. A sample of 1468 individuals with European ancestry were genotyped using the Illumina 610-Quad BeadChip (BHS1) and subsequent genotyping was carried out on an independent group of 3407 individuals with European ancestry using Illumina 660W-Quad (BHS2). Spirometric measures of FEV_1_ and FVC were assessed. From 1966 to 1978 (five surveys), FEV_1_ and FVC were measured using a McDermott dry spirometer (Pneumoconiosis Research Unit, Penarth, UK) and recorded as the highest values from three measurements (provided that two recordings were within 10% of each other). Wedge spirometers (Vitalograph, Buckingham, UK) were used in the 1981 survey and pneumotachograph spirometers (Welch Allyn, Skaneateles Falls, New York, USA) were used in 1994/1995.^11^ ^12^ FEV_1_ and FVC were measured in 2005 using Medgraphics pneumotachograph spirometers and recorded with BreezeSuite 6.2 (Medical Graphics, St Paul, USA).^13^ From 1981 onwards, spirometric measurements met American Thoracic Society guidelines (52% of the total number of measurements).^14^ ^15^

Demographic details and mean lung function measurements for this and replication cohorts are shown in table 1. Observations prior to age 25 were excluded from both discovery and replication analyses.^16^

**Table 1:** Descriptive characteristics of cohorts included in discovery and follow-up (age and lung function measurements as at first time point)

Study	No. of participants	Women, %	Ever-smokers, %	Mean age, years (SD)	Age range, years	Mean FEV_1_, L (SD)	Mean FVC, L (SD)	Mean FEV_1_/FVC (SD)	Mean annual change in FEV_1_, mL (SD)	Range of annual change in FEV_1_, mL
BHS	4167	56.1	48.8	38.6 (10.6)	25–82	3.35 (0.87)	4.26 (1.04)	0.79 (0.08)	−27.2 (20.3)	−150 to 90
CCHS	9016	55.6	75.7	46.4 (11.1)	25–85	2.98 (0.91)	3.69 (1.08)	0.81 (0.09)	−30.1 (29.7)	−260 to 220
LHS	3502	36.6	100	48.6 (6.7)	35–62	2.77 (0.63)	4.3 (0.94)	0.65 (0.07)	−51.0 (55.6)	−338 to 168

BHS, Busselton Health Study; CCHS, Copenhagen City Heart Study; LHS, Lung Health Study.

### Genotype quality control and imputation

Genotype data from BHS1 and BHS2 samples were merged and quality control (QC) was undertaken for both variant quality and sample quality. Prior to imputation, variants were excluded if they had a call rate <95%, deviated from Hardy-Weinberg equilibrium (p<10^−6^) or had a minor allele frequency (MAF) of <1% (100 240 variants). Individuals were excluded if their call rate was <95%, if their submitted gender and gender inferred by genotype were inconsistent, if they were a duplicate or if they were an outlier for heterozygosity (169 individuals). Principal components analysis was used to exclude individuals of non-European ancestry (those at least four SDs away from the mean for at least one of the first two principal components) (27 individuals). Analysis of identity by descent (IBD) was compared with the reported pedigree; any inconsistencies were reviewed and individuals were excluded as appropriate (40 individuals; see online supplementary material for further details). QC was undertaken using PLINK V.1.07 (Purcell S; http://pngu.mph.harvard.edu/purcell/plink),^17^ EIGENSOFT (V.4.2)^18^ ^19^ and R V.2.15 (R Core Team; http://www.R-project.org).

10.1136/thoraxjnl-2016-208448.supp1 supplementary data

Imputation was undertaken using the 1000 Genomes Project reference panel (1000G Phase I Integrated Release V.3 (March 2012)).^20^ Chunking was performed using ChunkChromosome, phasing using MaCH (V.1.0.18)^21^ and imputation using Minimac (V.2012.10.3).^22^ Variants where imputation quality (r^2^) was <0.3 or MAF <1% were excluded.

### Phenotype QC and analysis

Data on age and smoking were checked for consistency over time. Where possible, missing data on height or smoking at one time point were imputed to be the same as that at the nearest time point (407 data items imputed in total). Otherwise, observations with missing data on lung function, age or height or where FEV_1_ was greater than FVC were excluded from the analysis. After phenotype and genotype datasets were merged, and after samples failing genotype QC were excluded, 12 863 observations for 4170 individuals and up to eight time points were available prior to further phenotype QC.

A time variable was created for each observation, determined by the difference between the age of the individual at that observation and their age at the first time point for which data were available on that individual. Family was defined based on IBD, such that all individuals in a family were related (IBD >0.2) with at least one other person in the family. A full model with age, age^2^, height, height^2^, sex and time as fixed effects and an intercept varying per individual, an intercept varying per family and a slope for time varying per individual as random effects was fitted to the data for each trait (FEV_1_, FEV_1_/FVC and FVC). Additionally, another four models were fitted, excluding the random intercept varying per family, the random slope for time varying per individual and age^2^ and height^2^ one at a time and comparing with the full model using Akaike information criterion and Bayesian information criterion. The final model included all the terms in the full model, except for age^2^ as this did not improve the fit of the model (see online supplementary table S1).

Outlying observations (with residuals more than four SDs away from the mean) for any of the three traits analysed, FEV_1_, FEV_1_/FVC and FVC, were then excluded from the analysis (168 observations and three individuals excluded). This resulted in 12 695 observations for 4167 individuals and up to eight time points included in the final analysis. The mean number of measurements per participant was three (see online supplementary figure S1) and the mean length of follow-up was 15.5 years.

### Genetic risk scores

Previously published studies identified variants in 26 regions significantly associated with cross-sectional FEV_1_ and/or FEV_1_/FVC.^5–8^ We compared the effect size estimates for these 26 variants in BHS with the previously published effect size estimates. A weighted risk score was calculated for the joint effect of these 26 regions on baseline FEV_1_ and FEV_1_/FVC as well as on change over time in both traits. The single-nucleotide polymorphisms (SNPs) included in this analysis are shown in the online supplementary table S2.

Unbiased (winner’s curse-free) effect sizes, as calculated previously but excluding any data from BHS,^8^ were used as weights for the 26 variants in the risk score calculations (weights, range 0.2–2.57, provided in online supplementary table S3). The number of risk alleles for each variant was multiplied by its corresponding weight and then summed across variants in order to obtain the risk score for each individual and to create the risk score variable. This risk score variable was then added to the final model described above, together with the risk score by time interaction, in order to obtain both the effect on baseline and on change over time.

### Genome-wide association analysis and selection of variants for follow-up analysis

The effect of individual genetic variants (which included SNPs and indels) on the rate of decline in FEV_1_, FEV1/FVC and FVC was tested by adding a variant and variant-by-time interaction into the final phenotypic model described above. Using this model, genome-wide association analyses were undertaken for 29 798 550 variants assuming an additive genetic effect. Genomic control was subsequently applied (genomic inflation factors for slope change were 1.04, 1.03 and 1.06 for FEV_1_, FEV_1_/FVC and FVC, respectively).^23^

**Figure 1:**
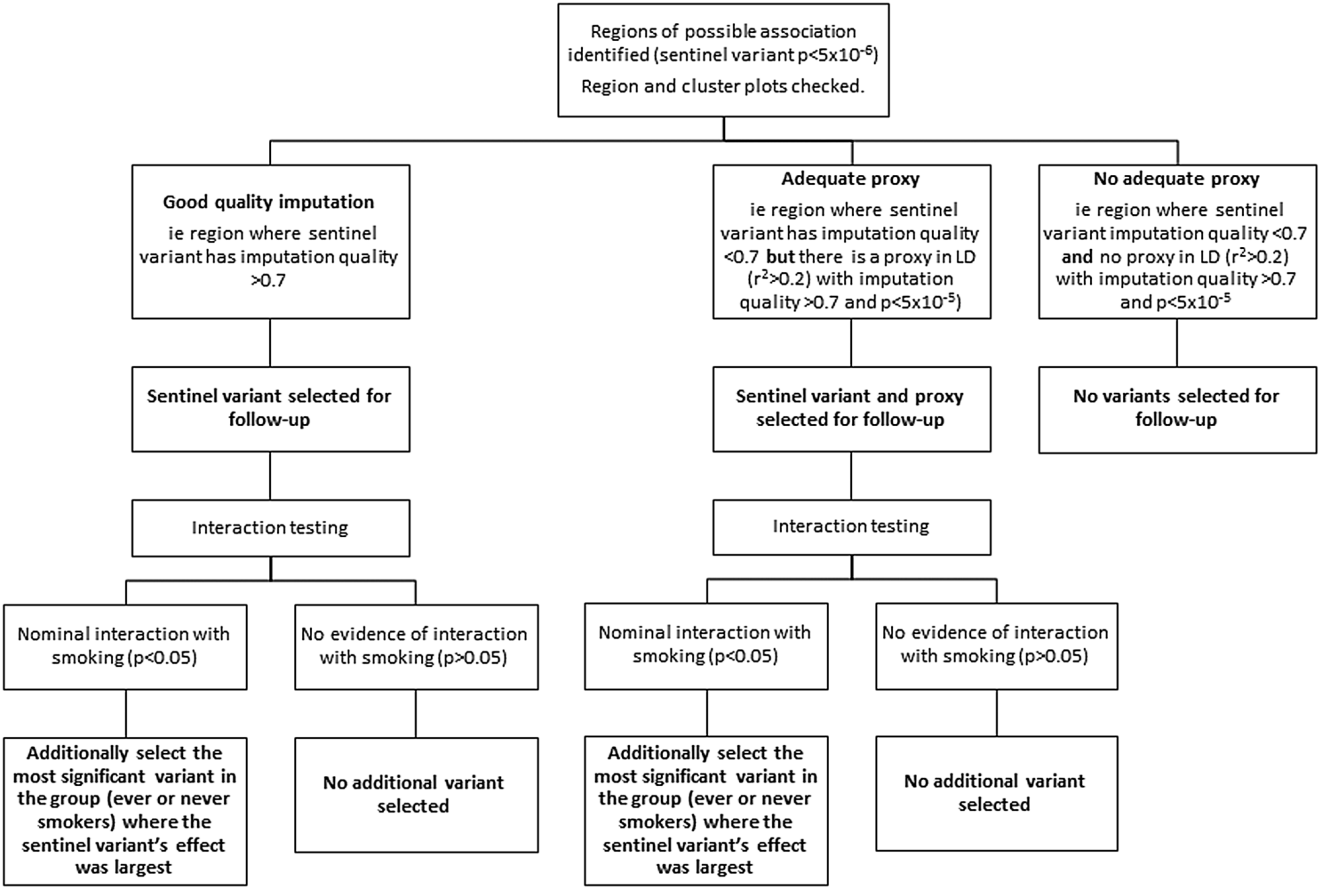
Flow chart showing the selection of variants for follow-up analysis. LD, linkage disequilibrium.

Figure 1 illustrates our criteria for selection of variants for follow-up analysis. We identified variants potentially associated (p<5×10^−6^ taken as suggestive of association) with longitudinal FEV_1_, FEV_1_/FVC or FVC. We defined regions of association around the most strongly associated variant (sentinel variant) ±500 kb. We examined region plots to assess support from neighbouring variants and cluster plots for the closest genotyped variant to the sentinel variant in order to rule out associations driven by genotyping errors. Regions were selected for follow-up if they had at least one variant (either the top variant or a proxy in linkage disequilibrium with r^2^ >0.2) with imputation quality >0.7 and p value <5×10^−5^.

Within each region, we selected variants for follow-up analysis as follows: (1) the sentinel variant, (2) a second variant in the region with imputation quality >0.7 and p value <5×10^−5^ where the sentinel variant had imputation quality <0.7 and (3) for regions in which variants showed nominal interaction with smoking (p <0.05 for interaction term based on a Z-test between ever-smokers and never-smokers), the most significant variant in the group (ever-smokers or never-smokers) where the sentinel variant's effect size estimate was largest.

Sensitivity analyses were also undertaken for the regions which met the criteria for follow-up analysis to assess whether their effect could be mediated through smoking behaviour. The same model was fitted, with an additional term for smoking status (ever-smoked or never-smoked), and effect sizes were compared.

Estimations of power were also obtained for the discovery of new signals and for detection of associations with the 26 variants previously reported to be associated with cross-sectional lung function (see online supplementary materials).

### Replication

Follow-up analyses for the 39 new regions potentially associated with longitudinal change were undertaken in the Copenhagen City Heart Study (CCHS), a prospective study of a random sample of the Danish general population, aged ≥20 years, drawn using the Danish Civil Registration System (n=9016) who had lung function measurements at up to three time points between 1976 and 1994,^24^ with a further measurement in 2001–03.^25^ Follow-up was also undertaken in the Lung Health Study (LHS), a North American cohort of smokers with mild airflow limitation who had annual lung function measurements for 5 years (n=3502).^26^ Risk score analyses with the previously reported 26 variants were also undertaken in CCHS.^5–8^ QC procedures (including inspection of cluster plots) were applied and the same model was fitted as for BHS, but without adjustment for relatedness, given that there were no related individuals. Demographic details and mean lung function measurements for CCHS and LHS are shown in table 1.

All risk score and association analyses were undertaken in R using the package ‘lme4’ (Bates D, Maechler M, Bolker B, *et al*; http://CRAN.R-project.org/package=lme4).

## Results

### Known regions: calculation of risk scores and replication

Comparison of effect sizes for cross-sectional lung function and longitudinal change and weighted risk score analyses showed that 26 variants previously identified as associated with cross-sectional lung function were not significantly associated with longitudinal change in our cohort.

**Figure 2:**
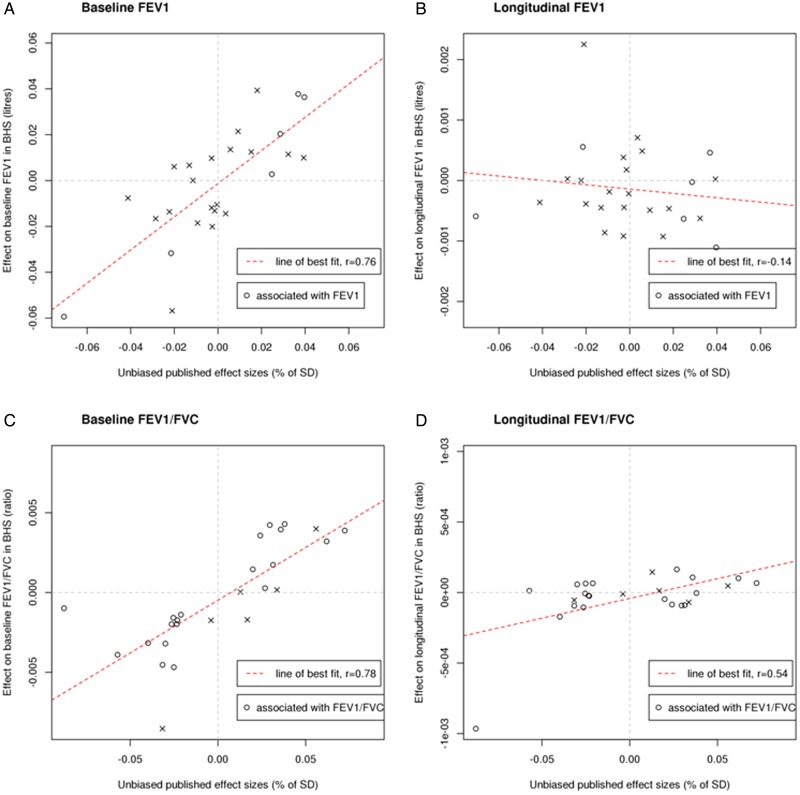
Representations showing the (A) correlation between estimated single-nucleotide polymorphism (SNP) effects on baseline FEV_1_ in Busselton Health Study (BHS) and published estimates, (B) correlation between estimated SNP effects on change in FEV_1_ in BHS and published estimates of SNP effects on cross-sectional FEV_1_, (C) correlation between estimated SNP effects on baseline FEV_1_/FVC in BHS and published estimates and (D) correlation between estimated SNP effects on change in FEV_1_/FVC in BHS and published estimates of SNP effects on cross-sectional FEV_1_/FVC (the correlation shown in (D) was reduced by excluding rs2070600, a non-synonymous coding SNP in *AGER* which had an outlying result (see online supplementary figure S2)). o, SNP significantly associated with cross-sectional measures of the trait under examination (FEV_1_ in (A) and (B); FEV_1_/FVC in (C) and (D)) in prior literature. x, SNP significantly associated with cross-sectional measures of the other trait (FEV_1_/FVC in (A) and (B); FEV_1_ in (C) and (D)) in prior literature.

There was a strong correlation between estimated SNP effects on baseline lung function in BHS and in published estimates^6–8^ for both FEV_1_ (r=0.76) and FEV_1_/FVC (r=0.78). However, estimated SNP effects on change in FEV_1_ and FEV_1_/FVC were weakly correlated with published estimates for the respective cross-sectional trait (figure 2 and online supplementary figure S2).

A weighted risk score was calculated for the joint effect of these 26 regions on baseline FEV_1_ and FEV_1_/FVC as well as change over time in both traits. This showed a strong effect on baseline FEV_1_ (p=9.75×10^−12^) and FEV_1_/FVC (p=4.44×10^−16^) but no effect was observed on change over time for either FEV_1_ (p=0.409) or FEV_1_/FVC (p=0.160) (table 2).

**Table 2:** Weighted risk score in Busselton Health Study (BHS) and Copenhagen City Heart Study (CCHS) for 26 known regions associated with FEV_1_ or FEV_1_/FVC

	BHS			CCHS		
	β	SE	p Value	β	SE	p Value
FEV_1_, mL (baseline)	−13.0	1.97	9.75×10^−12^	−6.10	1.24	8.92×10^−7^
FEV_1_, mL/year (change over time)	0.070	0.085	0.409	−0.13	0.058	0.030
FEV_1_/FVC (baseline)	−2.62×10^−3^	3.23×10^−4^	4.44×10^−16^	−1.28×10^−3^	2.05×10^−4^	4.45×10^−10^
FEV_1_/FVC (change over time)	−2.42×10^−5^	1.72×10^−5^	0.160	−1.37×10^−5^	1.33×10^−5^	0.302

β, per-allele change in FEV_1_ or FEV_1_/FVC.

In silico data were available for eight known variants in CCHS and genotyping was undertaken for the remaining 18 variants. All of these passed QC procedures. They showed a joint effect similar to that seen in BHS: a strong association with baseline measurement (p=4.45×10^−10^) but no association with change over time in FEV_1_/FVC (p=0.302) and strong association with baseline measurement (p=8.92×10^−7^) but only borderline association with change over time for FEV_1_ (p=0.030) (table 2).

### New signals: discovery and replication

Genome-wide analysis identified 56 independent regions which were associated with decline in FEV_1_, FEV_1_/FVC and/or FVC at a significance threshold of p<5x10^−6^. Thirty-nine of these regions (48 variants) were selected for follow-up based on the criteria described under ‘Methods’: 11 regions (13 variants) for change in FEV_1_, 15 regions (19 variants) for change in FVC and 13 regions (16 variants) for change in FEV_1_/FVC. These are shown in table 3. The most significantly associated variant (rs6502247) showed an effect of 3.93 mL/year on decline in FEV_1_ (p=3.23×10^−9^).

**Table 3:** Variants selected for follow-up and their association with longitudinal change in lung function (FEV_1_, FVC and FEV_1_/FVC) in Busselton Health Study (BHS)

Variant	Top variant in region	Chr	Position	Risk allele	Other allele	Risk allele frequency	β	SE	p Value
*(a) FEV_1_ (mL/year)*									
rs114969412	rs114969412	1	106234189	G	A	0.06	−4.84	1.00	1.29×10^−6^
rs13099788	rs13099788	3	192286178	C	G	0.56	−2.29	0.488	2.75×10^−6^
rs3819182	rs3819182	4	101107664	A	C	0.40	−2.14	0.460	3.47×10^−6^
rs3113685	rs3113685	4	109982140	G	T	0.39	−2.71	0.571	1.98×10^−6^
rs876365	rs3113685	4	109989467	C	T	0.31	−2.19	0.483	5.65×10^−6^
5:51298598:T_TG	5:51298598:T_TG	5	51298598	I	R	0.05	−6.19	1.32	2.97×10^−6^
rs76575180	5:51298598:T_TG	5	51546441	G	T	0.03	−6.72	1.61	3.15×10^−5^
5:136298545:A_AG	5:136298545:A_AG	5	136298545	I	R	0.88	−3.16	0.691	4.91×10^−6^
rs72901148	rs72901148	6	79562115	A	G	0.02	−8.95	1.73	2.15×10^−7^
rs11158759	rs11158759	14	69162043	C	T	0.84	−2.87	0.627	4.73×10^−6^
rs6502247	rs6502247	17	13203562	G	A	0.87	−3.93	0.663	3.23×10^−9^
rs8073053	rs8073053	17	19553382	A	G	0.30	−2.38	0.518	4.41×10^−6^
rs8132156	rs8132156	21	47062729	A	G	0.10	−4.17	0.833	5.72×10^−7^
rs113301658	rs113301658	22	34148991	C	G	0.14	−4.57	0.826	3.21×10^−8^
*(b) FVC (mL/year)*									
1:44578970:G_GCA	1:44578970:G_GCA	1	44578970	R	I	0.98	−10.3	2.10	1.07×10^−6^
rs878118	rs878118	3	71246228	T	G	0.80	−3.39	0.727	3.11×10^−6^
rs150801948	rs150801948	6	15920550	T	C	0.02	−10.0	2.16	3.46×10^−6^
rs111605394	rs111605394	7	51412802	A	T	0.96	−9.11	1.78	2.91×10^−7^
8:105858623:TTTC_	8:105858623:TTTC_	8	105858623	R	D	0.19	−3.84	0.818	2.71×10^−6^
9:424077:TA_T	9:424077:TA_T	9	424077	D	R	0.01	−14.8	3.09	1.70×10^−6^
rs118036814	rs118036814	11	107316060	A	G	0.07	−6.43	1.26	3.59×10^−7^
13:94098659:CT_C	13:94098659:CT_C	13	94098659	D	R	0.24	−3.32	0.701	2.18×10^−6^
rs62028012	rs62028012	15	97946036	C	T	0.73	−3.55	0.751	2.26×10^−6^
rs1862844	rs6539968	16	86758079	C	A	0.61	−4.64	0.878	1.24×10^−7^
rs6539968	rs6539968	16	86759924	C	T	0.64	−2.90	0.614	2.30×10^−6^
18:39303165:GTAGA	18:39303165:GTAGA	18	39303165	R	D	0.92	−5.21	1.13	4.35×10^−6^
rs12965811	rs12965811	18	63173334	T	G	0.07	−5.78	1.19	1.16×10^−6^
rs113179796	rs10404081	19	22748391	C	T	0.95	−12.0	2.21	5.17×10^−8^
rs10404081	rs10404081	19	22756773	A	G	0.97	−8.97	1.95	4.25×10^−6^
rs10404632	rs10404081	19	22763493	T	C	0.96	−6.23	1.49	2.89×10^−5^
rs2740192	rs2740185	20	3059396	C	T	0.14	−4.33	0.976	8.98×10^−6^
rs2740185	rs2740185	20	3061436	A	G	0.20	−4.35	0.930	3.00×10^−6^
*(c) FEV_1_/FVC (change per year)*									
rs11694877	rs11694877	2	5992517	G	T	0.91	−6.43×10^−4^	1.39×10^−4^	3.79×10^−6^
rs72847294	rs72847294	2	88684174	G	A	0.07	−7.90×10^−4^	1.52×10^−4^	2.14×10^−7^
rs6434439	rs6434439	2	191997584	A	C	0.43	−3.86×10^−4^	8.43×10^−5^	4.57×10^−6^
rs73832306	rs73832306	4	99370515	T	C	0.97	−1.13×10^−3^	2.48×10^−4^	4.90×10^−6^
rs10043201	rs116563943	5	1600242	G	A	0.77	−4.57×10^−4^	1.01×10^−4^	6.03×10^−6^
rs116563943	rs116563943	5	1606844	G	T	0.82	−6.74×10^−4^	1.45×10^−4^	3.26×10^−6^
rs58140608	rs114704427	5	170929096	C	T	0.93	−7.19×10^−4^	1.66×10^−4^	1.43×10^−5^
rs114704427	rs114704427	5	170941277	C	T	0.98	−1.83×10^−3^	3.54×10^−4^	2.18×10^−7^
rs512976	rs512976	6	7163873	T	C	0.15	5.29×10^−4^	1.14×10^−4^	3.20×10^−6^
rs11136718	rs11136718	8	4157216	G	A	0.63	−4.22×10^−4^	9.19×10^−5^	4.27×10^−6^
rs4737863	rs4737863	8	68857698	G	C	0.45	−3.71×10^−4^	7.89×10^−5^	2.56×10^−6^
rs72671203	rs72671203	14	20673455	G	A	0.13	−5.72×10^−4^	1.18×10^−4^	1.20×10^−6^
rs17197324	rs17197324	14	22079359	C	G	0.83	−4.94×10^−4^	1.03×10^−4^	1.80×10^−6^
rs117466318	rs117466318	14	82200378	A	G	0.97	−1.17×10^−3^	2.56×10^−4^	4.60×10^−6^
rs80245972	rs117466318	14	82205915	C	T	0.92	−5.80×10^−4^	1.43×10^−4^	4.72×10^−5^
rs16960347	rs16960347	17	64889056	C	T	0.93	−7.21×10^−4^	1.58×10^−4^	4.83×10^−6^

Betas provided correspond to the risk allele. Risk allele here is defined as the allele associated with decreased lung function in BHS.

Variants are given in order of Chr and position.

β, per-allele change in FEV_1_, FVC or FEV_1_/FVC; Chr, chromosome.

Effect size estimates for these variants were not attenuated after adjusting for smoking status and no variant-by-smoking interaction met a Bonferroni-corrected threshold for the number of regions tested (p<1.3×10^−3^).

De novo genotyping of all 48 variants was undertaken in CCHS. Where genotyping failed, tags were identified, if possible. In total, 34 variants in 30 independent regions passed QC procedures (including the inspection of cluster plots) and 14 failed. The analysis included 9016 individuals with up to four time points (25 796 observations). None of these 34 variants showed replicated association with change in lung function (using a significance threshold of p<0.0016, representing a Bonferroni correction for 30 independent regions with α=0.05) (see online supplementary table S4).

Thirty-one variants (in 26 of the 39 regions) for which in silico data were available were also followed up in LHS. None of these SNPs showed significant association with lung function decline in LHS (using a significance threshold of p<0.0019, representing a Bonferroni correction for 26 independent regions with α=0.05) (see online supplementary table S5). Results from both CCHS and LHS are shown in the online supplementary materials. In total, 21 variants were analysed in both follow-up studies, of these, 14 had the same direction of effect as BHS after meta-analysing results from CCHS and LHS (p=0.095).

For sentinel variants in regions previously reported to show suggestive evidence of association with longitudinal lung function (though none met genome-wide significance and replicated in previous papers), we assessed association with FEV_1_, FVC and/or FEV_1_/FVC in our data.^26–28^ Of these 51 variants,10 showed nominal evidence of association with at least one of the lung function traits either in the whole cohort or in one of the smoking subgroups. However, none were significant after correction for multiple testing (using a threshold of p<9.8×10^−4^) (see online supplementary table S6).

## Discussion

We analysed the joint effect of regions previously associated with cross-sectional lung function on longitudinal change in the general population, and undertook a GWAS to identify new signals associated with longitudinal change. Regions previously identified as significantly associated with cross-sectional FEV_1_ and/or FEV_1_/FVC^5–8^ were jointly strongly associated with baseline measurements in both discovery (BHS) and replication (CCHS) cohorts. However, although many of these variants have previously shown association with COPD risk (*TNS1*, *RARB*, *FAM13A*, *GSTCD*, *HHIP*, *HTR4*, *ADAM19*, *AGER*, *GPR126*, *C10orf11*, *THSD4*),^29–33^ we have shown that they are not associated with change in FEV_1_ or FEV_1_/FVC over time in our cohorts. We identified novel variants associated with decline in lung function in BHS which did not replicate in either a general population cohort (CCHS) or a cohort of smokers with mild lung function impairment (LHS).

A key strength of our study design is the improved coverage of both common and low-frequency variants achieved through imputation in the 1000 Genomes Project reference panel,^20^ compared with previous studies of longitudinal lung function which have used HapMap reference panels.^26^ ^27^ ^34^ Another strength is the high number of lung function measurements in BHS: up to eight measurements over a range of up to 40 years. This is longer than other published studies, including most of the individual studies in a meta-analysis by Tang *et al*,^34^ which included a portion of our dataset. Our study also adds value as it is the first to calculate risk scores for longitudinal lung function. Nevertheless, such a long follow-up period brings some challenges. Spirometry equipment changed over time and the earlier surveys in BHS were performed before protocols for standardisation of spirometry were published.^11^ ^14^ ^15^ In addition, repeated measurements over a number of years could train participants in optimal technique and underestimate decline in lung function. The detection of known associations with cross-sectional lung function provides some reassurance regarding the extent of any potential measurement error in lung function measurement.

The biggest challenge we faced was the availability of large sample sizes for well-characterised longitudinal measures of lung function. The sample size, in combination with possible measurement error, may have limited our ability to identify individual variants which reach stringent genome-wide significant levels and replicate. However, our study will have had much greater power to detect longitudinal effects of aggregate risk scores comprised of the 26 variants previously reported to be associated with cross-sectional lung function. We did not examine risk scores for FVC, as at the time of the analyses there were no published associations with FVC, and our focus was on the determinants of obstructive lung disease. It is possible therefore that there are genetic associations with change in FVC over time which we did not identify. We did not undertake analyses stratified by sex, for reasons of power. However, effect estimates were adjusted for sex. We did not adjust for smoking in the primary analysis, since the analysis also had potential to highlight novel signals for smoking behaviour. We undertook sensitivity analyses to assess whether any top signals could be mediated by smoking behaviour. The signals were not attenuated after adjustment for smoking. However, this was based on binary smoking status and there remains potential for incomplete adjustment.

Our study—the first to our knowledge to examine risk scores for change in lung function over time—complements existing studies which have sought individual SNP associations,^26–28^ including a large meta-analysis of longitudinal lung function.^34^ The meta-analysis by Tang *et al*^34^ (concurrent with our study) incorporated data from 27 349 individuals from 14 population-based cohorts (including a subset of 1009 individuals from BHS) and identified evidence for two novel regions associated with rate of change in FEV_1_. However, these did not replicate in two further cohort studies (including LHS). The authors noted that the number of lung function measurements and duration of follow-up varied considerably between studies included in the discovery phase and may have affected their ability to detect associations. The failure to replicate novel associations may relate to lack of power or, given that the larger replication cohort in their study (LHS) was composed of smokers with mild COPD, may indicate that the genetic determinants of lung function decline in those with COPD differ from those in healthy individuals.^34^

Similarly, a GWAS examining change in FEV_1_% predicted (in a cohort of smokers with mild lung function impairment from LHS) identified two regions reaching genome-wide significance which did not replicate in four general population cohorts or in a cohort with moderate-to-severe COPD. The authors suggested that regions which modify the effect of cigarette smoke on lung function decline (or determine rate of decline at different stages of COPD) may be distinct from those which influence lung function decline in the general population.^26^ A small number of studies have begun to explore interaction between genetic variants and smoking in relation to lung function decline, though these have not identified any significant associations which also replicated.^35^ ^36^

The suggestion that variants which determine lung function decline may show heterogeneity across different groups is further supported by findings from an earlier GWAS which identified suggestive evidence that different regions were associated with lung function decline in asthmatic and non-asthmatic individuals. Only the signal in non-asthmatic individuals (rs9316500 in *DLEU7*) showed evidence of replication (at p<0.05) in largely population-based cohorts, but it did not reach genome-wide significance in discovery, replication or two-stage meta-analysis.^27^ None of the top variants reported in these papers reached significance after correction for multiple testing in our data.

Our findings are also consistent with recent work by Lange *et al*^37^ which identified two distinct trajectories of FEV_1_ in people who developed COPD. In their study, approximately half of those diagnosed with COPD by the end of follow-up started with normal lung function in early adulthood (mean age 40) and then showed rapid decline, whereas the remaining half started with low FEV_1_ in early adulthood followed by a relatively normal rate of decline. This suggests that rapid lung function decline in later life is not necessary for development of COPD. We hypothesise that the known genetic variants examined in this paper may exert much of their effect in earlier life. Of the 26 regions examined in this paper, 19 have previously been shown to have directions of effect on lung function in children (aged 7–9 years) consistent with that in adults.^8^ An additional study has also shown evidence of association with lung function as early as 5–14 weeks of age for variants in 4 of the 26 loci.^38^ However, further large GWAS of lung development are required to test this hypothesis.

These findings emphasise the continuing public health importance of focusing on the key environmental determinants of lung function decline, particularly smoking. Nevertheless, genetic determinants of decline may remain a therapeutic target in the half of people with COPD for whom accelerated decline is important in pathogenesis.^37^ A potential strategy to identify these would be to focus on older cohorts and adjust for the effect of all known variants which affect cross-sectional lung function. Large sample sizes will also be the key to help confirm or refute our findings. However, as the largest existing meta-analysis identified significant challenges posed by phenotypic heterogeneity, ensuring comparability of the participating studies' approach to measuring longitudinal change must also be a high priority. An alternative approach would be to study longitudinal lung function in large, more homogeneous populations.

In summary, regions previously identified as significantly associated with cross-sectional FEV_1_ and/or FEV_1_/FVC^5–8^ were jointly strongly associated with baseline measurements in both discovery and replication cohorts but were not associated with change in FEV_1_ or FEV_1_/FVC over time. The present study and others to date have identified no regions associated with lung function decline which reach genome-wide significance and replicate in independent cohorts. Genetic variants identified to date that influence cross-sectional lung function, while still relevant in predicting the risk of COPD, appear to have little or no effect on the rate of change in adult lung function over time in our study.
