# Supplementary material for: Genetic variants affecting cross-sectional lung function in adults show little or no effect on longitudinal lung function decline
Source: Thorax. 2017 Feb 7;72(5):400–8. doi: 10.1136/thoraxjnl-2016-208448 (PMC5520280; doi:10.1136/thoraxjnl-2016-208448)
Supplement: supplementary data [file thoraxjnl-2016-208448supp001.pdf]

Supplementary Materials

The Busselton Health Study genetics study was approved by the University of Western Australia Human Ethics Committee under project numbers RA/4/1/1516 and RA/4/1/2077.

The Copenhagen City Heart Study genetic study was approved by Herlev and Gentofte Hospital, Copenhagen University Hospital, Denmark, by Danish ethical committees (the Copenhagen and Frederiksberg committee and the Copenhagen County committee; KF-100.2039/91, KF-01-144/01, H-KF-01-144/01).

The Lung Health Study protocols were approved by the institutional review board for human studies at each clinical centre, and written informed consent was obtained from each participant.

Supplementary Methods

Genotype quality control: exclusions based on identity-by-descent

40 individuals were excluded due to inconsistencies between the reported pedigree and the analysis of identity-by-descent (IBD). Reasons for exclusion were:

- where two individuals had IBD of 1 but inconsistent reported age (n=27);
- where IBD analysis indicated a pedigree inconsistent with the reported age (for example, a child older than their parent) (n=3);
- where there were inconsistencies in the IBD information for reported mothers/offspring (n=8);
- where IBD analysis indicated a pedigree which corresponded to the reported pedigree of a different individual (n=2).

Phenotype quality control and analysis

Full model (FM) (for *i*th individual in *j*th family at time point *k*):

$$Pheno_{ijk} = (\beta int + \beta int_{ij}) + \beta age\ x\ age_{ij} + \beta age2\ x\ age\ _{ij}{}^2 + \beta height\ x\ height_{ijk} + \beta height\ x\ height_{ijk}{}^2 + \beta sex\ x\ sex_{ij} + (\beta time + \beta time_{ij})\ x\ time_{ijk} + Eijk$$

$$with\ \beta int_{ij} = \beta intIND_{ij} + \beta intFAM_j$$

$$\beta intIND_{ij} \sim N(0, I\sigma_{IND}{}^2)$$
$$\beta intFAM_j \sim N(0, I\sigma_{FAM}{}^2)$$
$$\beta time_{ij} \sim N(0, I\sigma_{time}{}^2)$$

Table S1. Akaike Information Criterion (AIC) and Bayesian Information Criterion (BIC) for full and alternative models

	FEV_1		FEV_1/FVC		FVC	
	AIC	BIC	AIC	BIC	AIC	BIC
Full Model	11699	11781	-32238	-32156	14137	14220
FM without $\beta intFAM_j$	11829	11829	-32219	-32144	14219	14294
FM without $\beta time_{ij}$	12038	12105	-32192	-32125	14852	14919
FM without $age\ _{ij}{}^2$	11683	11757	-32255	-32181	14149	14224
FM without $height\ _{ijk}{}^2$	11757	11832	-32231	-32156	14177	14252

FEV$_1$, forced expiratory volume in one second. FVC, forced vital capacity. FEV$_1$/FVC, ratio of forced expiratory volume in one second to forced vital capacity.

Final model (for i^{th} individual in j^{th} family at time point k):

$$Pheno_{ijk} = (\beta int + \beta int_{ij}) + \beta age\ x\ age_{ij} + \beta height\ x\ height_{ijk} + \\ + \beta height\ x\ height_{ijk}{}^{2} + \beta sex\ x\ sex_{ij} + (\beta time + \beta time_{ij})\ x\ time_{ijk} + E_{ijk}$$

$$with\ \beta int_{ij} = \beta intIND_{ij} + \beta intFAM_{j}$$

$$\beta intIND_{ij} \sim N(0, I\sigma_{IND}{}^{2})$$
$$\beta intFAM_{j} \sim N(0, I\sigma_{FAM}{}^{2})$$
$$\beta time_{ij} \sim N(0, I\sigma_{time}{}^{2})$$

Figure S1. Distribution of lung function measurements in BHS

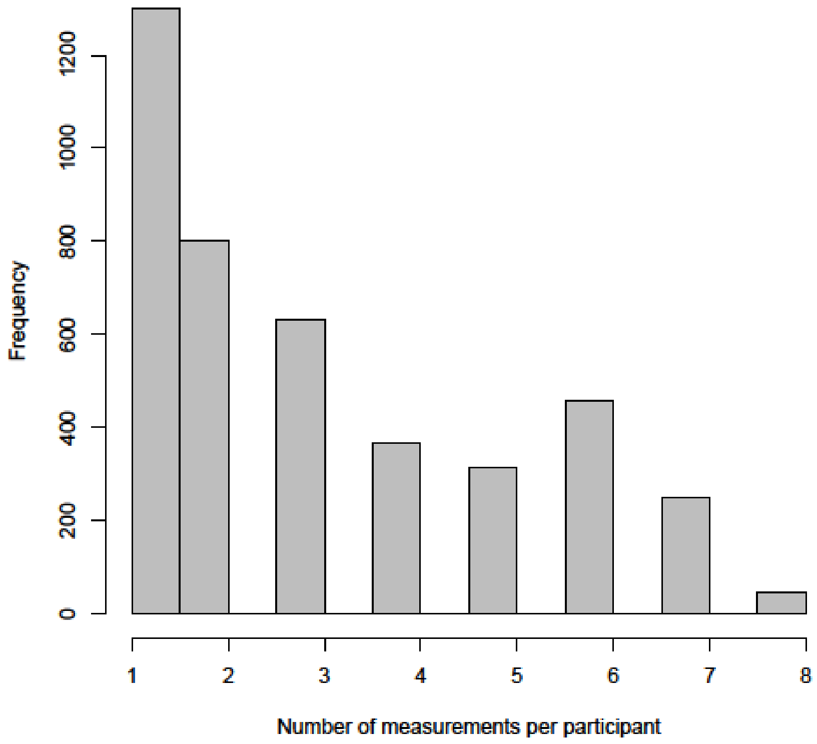

Power calculations

Power calculations were obtained by running 100 simulations in a simplified scenario of 1836 unrelated individuals (the number of families included in the analysis) and 3 time points for each individual (the mean number of time points per individual). A mixed model was fitted with time, SNP and SNP-by-time interaction (as fixed effects), an intercept varying per individual and a slope for time varying per individual (as random effects). The intercept, the effect of time and the variance of the random intercept, random slope for time and random error were extracted from the model used in the analysis for FEV$_1$. The effect of SNP on baseline was assumed to be 0 for simplicity.

In order to achieve power >80% for FEV_1 in the discovery GWAS using a significance threshold of $5x10^{-6}$ (follow-up threshold), effect sizes >= 93%, 41% and 20% of the standard deviation (SD) of the phenotypic rate of decline would be required for variants with MAF 1%, 5% and 40% respectively. Based on the SD observed in BHS for annual change in FEV_1, this would equate to effect sizes between 4.1ml/yr and 18.9ml/yr.

In order to achieve power >80% to detect associations with the 26 known variants for FEV_1 (using a significance threshold of 0.0019, a Bonferroni correction for 26 tests), we would need effect sizes >30% of SD for MAF=5% (pertinent to one of the 26 known signals), >24% of SD for 10%<MAF<15% (pertinent to 7 of the 26 known signals), >17% of SD for 20%<MAF<35% (pertinent to 9 signals) and >14% of SD to detect 40%<MAF<50% (pertinent to 9 signals). Based on the SD observed in BHS for annual change in FEV_1, this would equate to effect sizes between 2.8ml/yr and 6.1ml/yr.

Supplementary Results

Figure S2. Correlation between estimated SNP effects on change in FEV_1/FVC in BHS and published estimates of SNP effects on cross-sectional FEV_1/FVC after exclusion of outlying SNP rs2070600

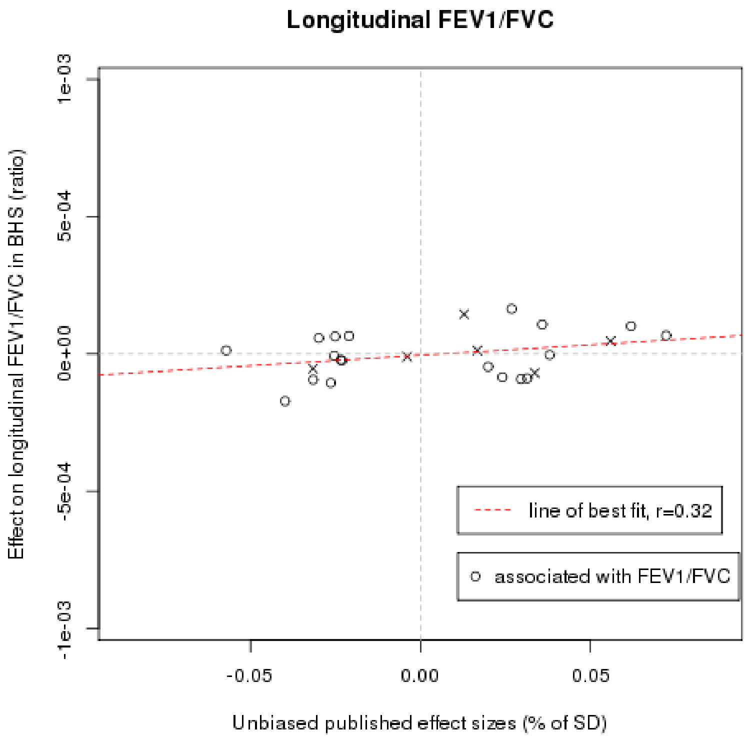

o, SNP significantly associated with cross-sectional measures of FEV_1/FVC in prior literature.
x, SNP significantly associated with cross-sectional measures of FEV_1 in prior literature.
FEV_1/FVC, ratio of forced expiratory volume in one second to forced vital capacity.

Table S2. SNPs included in risk score analysis and their individual effect sizes on longitudinal change in FEV_1 and FEV_1/FVC

Variant	Chr	Pos	Gene	Alleles	Risk allele	FEV_1, ml/yr			FEV_1/FVC, change per year		
						Beta	SE	P	Beta	SE	P
rs2284746	1	17306675	MFAP2	C/G	G	-0.179	0.451	0.692	$3.64x10^{-6}$	$7.81x10^{-5}$	0.963
rs993925	1	218860068	TGFB2	C/T	C	-0.445	0.473	0.347	$-2.45x10^{-5}$	$8.22x10^{-5}$	0.766
rs2571445	2	218683154	TNS1	A/G	A	1.11	0.461	0.016	$6.95x10^{-5}$	$8.02x10^{-5}$	0.386
rs12477314	2	239877148	HDAC4	C/T	C	0.026	0.541	0.961	$5.77x10^{-5}$	$9.40x10^{-5}$	0.539
rs1529672	3	25520582	RARB	A/C	C	-0.449	0.583	0.441	$-9.40x10^{-5}$	$1.01x10^{-4}$	0.354
rs1344555	3	169300219	MECOM	C/T	T	0.633	0.575	0.271	$-1.23x10^{-5}$	$1.00x10^{-4}$	0.902
rs2045517	4	89870964	FAM13A	C/T	T	-0.487	0.445	0.274	$-1.07x10^{-4}$	$7.72x10^{-5}$	0.166
rs10516526	4	106688904	GSTCD	A/G	A	-0.591	0.894	0.508	$-5.40x10^{-5}$	$1.55x10^{-4}$	0.728
rs11100860	4	145479139	HHIP	A/G	A	-0.362	0.451	0.422	$1.25x10^{-5}$	$7.83x10^{-5}$	0.873
rs153916	5	95036700	SPATA9	C/T	T	0.709	0.440	0.107	$-6.75x10^{-6}$	$7.63x10^{-5}$	0.930
rs1985524	5	147847788	HTR4	C/G	G	-0.461	0.449	0.305	$-4.69x10^{-5}$	$7.82x10^{-5}$	0.548
rs11134779	5	156936766	ADAM19	A/G	G	0.626	0.459	0.173	$8.49x10^{-5}$	$7.97x10^{-5}$	0.287
rs6903823	6	28322296	ZKSCAN3	A/G	G	0.027	0.517	0.959	$-1.45x10^{-4}$	$9.00x10^{-5}$	0.108
rs114327456	6	31568469	NCR3	A/G	A	0.464	0.573	0.417	$-1.64x10^{-4}$	$9.96x10^{-5}$	0.100
rs2070600	6	32151443	AGER	C/T	C	2.25	3.70	0.543	$-9.65x10^{-4}$	$6.37x10^{-4}$	0.130
rs2798641	6	109268050	ARMC2	C/T	T	0.490	0.558	0.379	$9.19x10^{-5}$	$9.66x10^{-5}$	0.342
rs262129	6	142853144	LOC153910	A/G	A	-0.862	0.469	0.066	$-1.72x10^{-4}$	$8.14x10^{-5}$	0.035
rs16909859	9	98204792	PTCH1	A/G	A	0.386	0.897	0.667	$-1.01x10^{-4}$	$1.55x10^{-4}$	0.515
rs7068966	10	12277992	CDC123	C/T	C	$1.14x10^{-3}$	0.436	0.998	$6.53x10^{-5}$	$7.56x10^{-5}$	0.387
rs11001819	10	78315224	C10orf11	A/G	G	0.556	0.438	0.204	$-1.04x10^{-5}$	$7.60x10^{-5}$	0.891
rs11172113	12	57527283	LRP1	C/T	T	-0.919	0.446	0.039	$-1.05x10^{-4}$	$7.75x10^{-5}$	0.174
rs1036429	12	96271428	CCDC38	C/T	C	-0.216	0.542	0.690	$6.36x10^{-5}$	$9.39x10^{-5}$	0.498
rs8033889	15	71680080	THSD4	G/T	T	-0.022	0.532	0.966	$-6.71x10^{-5}$	$9.24x10^{-5}$	0.468
rs12447804	16	58075282	MMP15	C/T	T	-0.383	0.533	0.472	$4.69x10^{-5}$	$9.25x10^{-5}$	0.612
rs2865531	16	75390316	CFDP1	A/T	A	-0.186	0.454	0.683	$-2.21x10^{-5}$	$7.92x10^{-5}$	0.780
rs9978142	21	35652239	KCNE2	A/T	T	0.926	0.62	0.135	$8.93x10^{-5}$	$1.07x10^{-4}$	0.405

Betas provided correspond to the risk allele. Risk allele here is defined as the allele that decreases lung function for the trait (FEV_1 or FEV_1/FVC) with smaller P-value as previously reported.[1]

FEV_1, forced expiratory volume in one second. FVC, forced vital capacity. FEV_1/FVC, ratio of forced expiratory volume in one second to forced vital capacity. Chr, chromosome. Pos, position. Beta, per-allele change in FEV_1 or FEV_1/FVC. SE, standard error.

Table S3. Weights included in the risk scores for FEV_1 and FEV_1/FVC

Variant	Chr	Pos	Gene	Alleles	Risk allele	FEV_1		FEV_1/FVC	
						Beta	Weight	Beta	Weight
rs1036429	12	96271428	CCDC38	C/T	C	-0.0004	0.01991	-0.02511	0.737512
rs10516526	4	106688904	GSTCD	A/G	A	-0.07056	3.510826	-0.03161	0.928437
rs11001819	10	78315224	C10orf11	A/G	G	-0.02147	1.068518	-0.0039	0.114456
rs11100860	4	145479139	HHIP	A/G	A	-0.04124	2.051909	-0.05715	1.678783
rs11134779	5	156936766	ADAM19	A/G	G	-0.03228	1.606352	-0.02412	0.708651
rs11172113	12	57527283	LRP1	C/T	T	-0.00288	0.143075	-0.02636	0.774297
rs114327456	6	31568469	NCR3	A/G	A	-0.01803	0.897226	-0.02685	0.788573
rs12447804	16	58075282	MMP15	C/T	T	0.002881	0.143349	-0.01991	0.584919
rs12477314	2	239877148	HDAC4	C/T	C	-0.02853	1.419381	-0.02992	0.878889
rs1344555	3	169300219	MECOM	C/T	T	-0.02475	1.231424	-0.01675	0.492136
rs1529672	3	25520582	RARB	A/C	C	-0.01311	0.652274	-0.03156	0.927054
rs153916	5	95036700	SPATA9	C/T	T	0.003575	0.177876	-0.02536	0.744968
rs16909859	9	98204792	PTCH1	A/G	A	0.020089	0.999594	-0.06195	1.819698
rs1985524	5	147847788	HTR4	C/G	G	-0.03682	1.831884	-0.05596	1.643688
rs2045517	4	89870964	FAM13A	C/T	T	-0.00573	0.285244	-0.03584	1.052637
rs2070600	6	32151443	AGER	C/T	C	-0.02099	1.044492	-0.08765	2.574695
rs2284746	1	17306675	MFAP2	C/G	G	0.001482	0.073767	-0.03803	1.117155
rs2571445	2	218683154	TNS1	A/G	A	-0.03966	1.973272	-0.03364	0.988079
rs262129	6	142853144	LOC153910	A/G	A	-0.01144	0.569193	-0.03981	1.169529
rs2798641	6	109268050	ARMC2	C/T	T	-0.00924	0.459862	-0.02958	0.868767
rs2865531	16	75390316	CFDP1	A/T	A	-0.00934	0.464512	-0.02343	0.688109
rs6903823	6	28322296	ZKSCAN3	A/G	G	-0.02859	1.422697	-0.01284	0.377194
rs7068966	10	12277992	CDC123	C/T	C	-0.02225	1.10725	-0.02102	0.617576
rs8033889	15	71680080	THSD4	G/T	T	-0.03933	1.956879	-0.07228	2.123216
rs993925	1	218860068	TGFB2	C/T	C	-0.00258	0.128467	-0.02308	0.677821
rs9978142	21	35652239	KCNE2	A/T	T	-0.01529	0.760769	-0.03143	0.923164

Betas were obtained as unbiased estimates (excluding any data involved discovery of each variant) as previously reported [1] but removing any BHS data and provided as the % of the standard deviation for FEV_1 and FEV_1/FVC. These betas were scaled so the weights add up to 26 and these weights were used in the risk score calculations. Betas provided correspond to the risk allele. Risk allele here is defined as the allele that decreases lung function for the trait (FEV1 or FEV1/FVC) with smaller P-value as previously reported.[1]

Table S4. Results of follow-up of 34 regions in CCHS

Trait	Top variant in region (BHS)	Variant (CCHS)	Chr	Pos	Risk allele	Other allele	Beta	SE	P
FEV_1, ml/yr	rs13099788	rs13099788	3	192286178	C	G	0.016	0.378	0.966
	rs3819182	rs3819182	4	101107664	A	C	0.383	0.384	0.318
	rs3113685	rs3113685	4	109982140	G	T	-0.612	0.409	0.135
	rs3113685	rs876365	4	109989467	C	T	-0.593	0.415	0.153
	5:51298598:T_TG	rs76575180	5	51546441	G	T	-0.312	0.978	0.750
	5:136298545:A_AG	5:136298545:A_AG	5	136298545	I	R	0.028	0.538	0.959
	rs72901148	rs72901148	6	79562115	A	G	-1.29	1.36	0.344
	rs6502247	rs6502247	17	13203562	G	A	0.229	0.569	0.687
	rs8073053	rs4646797	17	19560368	T	G	-0.235	0.449	0.600
	rs8132156	rs2330186	21	47052223	A	G	-0.300	0.660	0.650
FVC, ml/yr	rs878118	rs878118	3	71246228	T	G	-0.100	0.545	0.855
	rs150801948	rs150801948	6	15920550	T	C	-1.11	1.84	0.546
	rs111605394	rs111605394	7	51412802	A	T	-0.494	1.31	0.706
	8:105858623:TTTC_	8:105858623:TTTC_	8	105858623	R	D	-0.699	0.625	0.263
	rs118036814	rs11212236	11	107310162	A	G	-1.13	0.912	0.214
	13:94098659:CT_C	rs1373835	13	94094597	A	G	0.160	0.469	0.733
	rs62028012	rs62028012	15	97946036	C	T	-0.388	0.472	0.411
	rs6539968	rs1862844	16	86758079	C	A	-0.436	0.442	0.324
	rs6539968	rs6539968	16	86759924	C	T	-0.474	0.449	0.292
	18:39303165:GTAGA	rs16975198	18	39279863	C	T	0.034	0.773	0.965
	rs12965811	rs12956284	18	63180214	A	G	1.07	1.01	0.289
	rs10404081	rs10404081	19	22756773	A	G	0.596	1.02	0.559
	rs2740185	rs2740192	20	3059396	C	T	-0.643	0.584	0.271
	rs2740185	rs2740185	20	3061436	A	G	-1.01	0.578	0.080
FEV_1/FVC, change per year	rs11694877	rs11694877	2	5992517	G	T	-1.73×10^{-5}	1.43×10^{-4}	0.904
	rs72847294	rs72847294	2	88684174	G	A	9.66×10^{-6}	1.48×10^{-4}	0.948
	rs73832306	rs73832306	4	99370515	T	C	-3.86×10^{-4}	2.79×10^{-4}	0.165
	rs114704427	rs114704427	5	170941277	C	T	-7.94×10^{-5}	2.92×10^{-4}	0.786
	rs512976	rs512976	6	7163873	T	C	2.68×10^{-4}	1.06×10^{-4}	0.012
	rs4737863	rs4737863	8	68857698	G	C	6.87×10^{-5}	7.80×10^{-5}	0.378
	rs17197324	rs17197324	14	22079359	C	G	3.00×10^{-5}	1.10×10^{-4}	0.784
	rs117466318	rs117466318	14	82200378	A	G	-8.39×10^{-5}	2.05×10^{-4}	0.682
	rs117466318	rs80245972	14	82205915	C	T	-5.57×10^{-5}	1.48×10^{-4}	0.706
	rs16960347	rs16960347	17	64889056	C	T	2.00×10^{-4}	1.63×10^{-4}	0.220

Betas provided correspond to the risk allele. Risk allele here is defined as the allele that decreases lung function in the discovery stage (BHS).

FEV_1, forced expiratory volume in one second. FVC, forced vital capacity. FEV_1/FVC, ratio of forced expiratory volume in one second to forced vital capacity. Chr, chromosome. Pos, position. Beta, per-allele change in FEV_1 or FEV_1/FVC. SE, standard error.

Table S5. Results of follow-up of 26 regions in LHS

Trait	Top variant in region (BHS)	Variant (LHS)	Chr	Pos	Risk allele	Other allele	Beta	SE	P
FEV_1, ml/yr	rs114969412	rs114969412	1	106234189	G	A	-3.53	12.5	0.777
	rs13099788	rs13099788	3	192286178	C	G	-7.95	5.35	0.137
	rs3819182	rs3819182	4	101107664	A	C	-1.40	5.29	0.791
	rs3113685	rs3113685	4	109982140	G	T	3.90	6.10	0.523
	rs3113685	rs876365	4	109989467	C	T	5.00	5.36	0.351
	rs72901148	rs72901148	6	79562115	A	G	-13.3	19.6	0.497
	rs11158759	rs11158759	14	69162043	C	T	1.94	6.85	0.777
	rs6502247	rs6502247	17	13203562	G	A	12.4	7.65	0.104
FVC, ml/yr	rs878118	rs878118	3	71246228	T	G	-6.09	9.45	0.519
	rs111605394	rs111605394	7	51412802	A	T	-5.59	19.8	0.777
	rs118036814	rs118036814	11	107316060	A	G	17.7	15.6	0.255
	rs62028012	rs62028012	15	97946036	C	T	6.66	9.22	0.470
	rs6539968	rs1862844	16	86758079	C	A	9.44	7.42	0.203
	rs6539968	rs6539968	16	86759924	C	T	8.73	7.39	0.238
	rs12965811	rs12965811	18	63173334	T	G	2.77	15	0.854
	rs10404081	rs10404632	19	22763493	T	C	-10.5	23.3	0.651
	rs2740185	rs2740192	20	3059396	C	T	25.6	13.6	0.060
	rs2740185	rs2740185	20	3061436	A	G	21.5	12.9	0.095
FEV_1/FVC, change per year	rs11694877	rs11694877	2	5992517	G	T	$2.52x10^{-4}$	$4.36x10^{-4}$	0.563
	rs72847294	rs72847294	2	88684174	G	A	$2.29x10^{-4}$	$4.83x10^{-4}$	0.634
	rs73832306	rs73832306	4	99370515	T	C	$4.64x10^{-4}$	$8.59x10^{-4}$	0.589
	rs116563943	rs116563943	5	1606844	G	T	$-1.50x10^{-4}$	$4.92x10^{-4}$	0.760
	rs114704427	rs58140608	5	170929096	C	T	$9.18x10^{-4}$	$8.04x10^{-4}$	0.253
	rs114704427	rs114704427	5	170941277	C	T	$7.28x10^{-5}$	$1.60x10^{-3}$	0.964
	rs11136718	rs11136718	8	4157216	G	A	$-3.72x10^{-4}$	$3.30x10^{-4}$	0.260
	rs4737863	rs4737863	8	68857698	G	C	$-1.82x10^{-4}$	$2.68x10^{-4}$	0.498
	rs72671203	rs72671203	14	20673455	G	A	$-2.41x10^{-4}$	$3.49x10^{-4}$	0.490
	rs17197324	rs17197324	14	22079359	C	G	$-2.65x10^{-4}$	$3.58x10^{-4}$	0.458
	rs117466318	rs80245972	14	82205915	C	T	$5.00x10^{-4}$	$4.70x10^{-4}$	0.287
	rs16960347	rs16960347	17	64889056	C	T	$-3.34x10^{-4}$	$4.92x10^{-4}$	0.498

Betas provided correspond to the risk allele. Risk allele here is defined as the allele that decreases lung function in the discovery stage (BHS).
FEV_1, forced expiratory volume in one second. FVC, forced vital capacity. FEV_1/FVC, ratio of forced expiratory volume in one second to forced vital capacity. Chr, chromosome. Pos, position. Beta, per-allele change in FEV_1 or FEV_1/FVC. SE, standard error.

Table S6a. Lookup results in BHS for previously reported regions [whole cohort][2-4]

Paper	Top SNP in region in original paper	Associated trait (in original paper)	Cohort (in original paper)	P (in original paper)	Replicated (in original paper)	Coding/ non-coding allele	Freq	FEV1 (ml/yr)			FVC (ml/yr)			FEV1/FVC		
								Beta	SE	P	Beta	SE	P	Beta	SE	P
Hansel et al (2013)	rs7911302	FEV1 (% predicted)	COPD	**4.4x10^{-8} ***	No	C/A	0.578	0.483	0.442	0.275	0.308	0.574	0.592	7.77x10^{-5}	7.69x10^{-5}	0.312
Hansel et al (2013)	rs177852	FEV1 (% predicted)	COPD	**6.3x10^{-8} ***	No	C/T	0.664	0.533	0.472	0.259	0.859	0.614	0.162	2.58x10^{-5}	8.24x10^{-5}	0.754
Hansel et al (2013)	rs2030436	FEV1 (% predicted)	COPD	3.2x10^{-7}	No	A/G	0.868	-0.202	0.647	0.754	0.228	0.843	0.787	-1.44x10^{-4}	1.12x10^{-4}	0.200
Hansel et al (2013)	rs12194741	FEV1 (% predicted)	COPD	1.8x10^{-6}	No	T/C	0.789	0.121	0.529	0.819	-0.361	0.689	0.601	1.02x10^{-4}	9.17x10^{-5}	0.266
Hansel et al (2013)	rs2275456	FEV1 (% predicted)	COPD	3.1x10^{-6}	No	T/C	0.954	-0.179	1.035	0.863	-1.541	1.344	0.252	3.13x10^{-4}	1.81x10^{-4}	0.084
Hansel et al (2013)	rs7331728	FEV1 (% predicted)	COPD	3.6x10^{-6}	No	A/G	0.832	0.190	0.592	0.749	0.492	0.770	0.523	-8.57x10^{-5}	1.03x10^{-4}	0.405
Hansel et al (2013)	rs4330400	FEV1 (% predicted)	COPD	7.3x10^{-6}	No	G/A	0.376	0.111	0.451	0.806	0.030	0.588	0.959	2.49x10^{-6}	7.82x10^{-5}	0.975
Liao et al (2014)	rs1450439	FEV1	General population	4.38x10^{-6}	Not attempted	A/G	0.778	1.006	0.535	0.060	0.308	0.696	0.658	2.34x10^{-4}	9.29x10^{-5}	**0.012 ****
Liao et al (2014)	rs33794	FEV1	General population	6.07x10^{-6}	Not attempted	A/G	0.538	0.777	0.444	0.080	1.059	0.578	0.067	-2.97x10^{-5}	7.74x10^{-5}	0.701
Liao et al (2014)	rs10459348	FEV1	General population	9.84x10^{-6}	Not attempted	C/T	0.715	0.394	0.479	0.411	0.318	0.625	0.611	5.56x10^{-5}	8.29x10^{-5}	0.502
Liao et al (2014)	rs1349345	FEV1	General population	1.39x10^{-5}	Not attempted	C/G	0.645	0.839	0.471	0.075	0.892	0.613	0.146	6.84x10^{-5}	8.17x10^{-5}	0.403
Liao et al (2014)	rs7227539	FEV1	General population	1.58x10^{-5}	Not attempted	A/G	0.932	0.909	0.839	0.278	-0.897	1.093	0.412	4.45x10^{-4}	1.45x10^{-4}	**0.002 ****
Liao et al (2014)	rs9365009	FEV1	General population	1.98x10^{-5}	Not attempted	C/A	0.254	-0.622	0.503	0.216	0.274	0.656	0.676	-1.55x10^{-4}	8.73x10^{-5}	0.076
Liao et al (2014)	rs10779128	FEV1	General population	2.28x10^{-5}	Not attempted	A/G	0.775	1.259	0.524	**0.016 ****	1.719	0.683	**0.012 ****	1.75x10^{-6}	9.07x10^{-5}	0.985
Liao et al (2014)	rs17417768	FEV1/FVC	General population	1.15x10^{-5}	Not attempted	T/C	0.911	0.640	0.766	0.404	1.907	0.997	0.056	-8.20x10^{-5}	1.33x10^{-4}	0.538
Liao et al (2014)	rs7117082	FEV1/FVC	General population	1.37x10^{-5}	Not attempted	G/T	0.917	0.082	0.810	0.919	-0.138	1.055	0.896	1.36x10^{-4}	1.41x10^{-4}	0.332
Liao et al (2014)	rs11222969	FEV1/FVC	General population	1.66x10^{-5}	Not attempted	C/T	0.860	0.147	0.644	0.819	0.150	0.838	0.858	6.88x10^{-5}	1.12x10^{-4}	0.540
Liao et al (2014)	rs3760905	FEV1/FVC	General population	1.91x10^{-5}	Not attempted	G/T	0.829	0.032	0.667	0.962	-0.559	0.870	0.521	1.38x10^{-4}	1.15x10^{-4}	0.231
Liao et al (2014)	rs7167951	FEV1/FVC	General population	2.30x10^{-5}	Not attempted	T/G	0.708	-0.056	0.486	0.908	-0.303	0.633	0.633	2.44x10^{-5}	8.44x10^{-5}	0.773

Liao et al (2014)	rs7945975	FEV1/FVC	General population	2.45×10^{-5}	Not attempted	A/G	0.860	-0.782	0.640	0.222	-1.078	0.833	0.195	-9.77×10^{-6}	1.11×10^{-4}	0.930
Liao et al (2014)	rs7620133	FEV1/FVC	General population	2.49×10^{-5}	Not attempted	C/T	0.685	0.193	0.469	0.681	0.512	0.610	0.402	-4.41×10^{-5}	8.13×10^{-5}	0.588
Imboden et al (2012)	rs1889321	FEV1	General population	6.95×10^{-7}	No	T/C	0.738	0.394	0.492	0.423	1.508	0.641	**0.019 ****	-1.72×10^{-4}	8.51×10^{-5}	**0.044 ****
Imboden et al (2012)	rs9316500	FEV1	General population	4.81×10^{-6}	**Yes**	T/G	0.691	0.116	0.473	0.806	0.001	0.615	0.998	3.43×10^{-5}	8.22×10^{-5}	0.677
Imboden et al (2012)	rs6785065	FEV1	General population	1.00×10^{-5}	No	C/T	0.770	-0.253	0.515	0.624	-0.068	0.670	0.919	1.91×10^{-5}	8.96×10^{-5}	0.831
Imboden et al (2012)	rs278037	FEV1	General population	2.00×10^{-5}	No	C/T	0.845	-0.368	0.601	0.540	-0.302	0.784	0.700	-4.00×10^{-5}	1.04×10^{-4}	0.701
Imboden et al (2012)	rs7641198	FEV1	General population	3.00×10^{-5}	No	C/A	0.876	-0.189	0.665	0.777	0.655	0.867	0.450	-1.98×10^{-4}	1.15×10^{-4}	0.086
Imboden et al (2012)	rs421847	FEV1	General population	3.00×10^{-5}	No	G/A	0.725	0.360	0.496	0.467	-0.558	0.646	0.387	6.90×10^{-5}	8.61×10^{-5}	0.423
Imboden et al (2012)	rs496809	FEV1	General population	4.00×10^{-5}	No	C/T	0.943	-1.327	0.928	0.153	-1.250	1.205	0.299	-4.97×10^{-5}	1.62×10^{-4}	0.760
Imboden et al (2012)	rs10933964	FEV1	General population	6.00×10^{-5}	No	A/G	0.499	-0.625	0.463	0.177	-0.854	0.603	0.157	-4.12×10^{-6}	8.04×10^{-5}	0.959
Imboden et al (2012)	rs10808265	FEV1	Asthmatic	1.66×10^{-6}	No	G/A	0.548	-0.446	0.443	0.315	-0.642	0.577	0.266	3.42×10^{-5}	7.70×10^{-5}	0.657
Imboden et al (2012)	rs1902618	FEV1	Asthmatic	1.72×10^{-6}	No	A/G	0.810	-0.060	0.559	0.914	0.067	0.728	0.927	-1.13×10^{-5}	9.69×10^{-5}	0.907
Imboden et al (2012)	rs3843306	FEV1	Asthmatic	5.11×10^{-6}	No	T/A	0.563	-0.089	0.446	0.841	-0.438	0.580	0.451	-3.65×10^{-5}	7.73×10^{-5}	0.636
Imboden et al (2012)	rs7006290	FEV1	Asthmatic	5.19×10^{-6}	No	G/A	0.683	0.514	0.474	0.278	0.971	0.618	0.116	3.02×10^{-5}	8.20×10^{-5}	0.712
Imboden et al (2012)	rs12436689	FEV1	Asthmatic	6.87×10^{-6}	No	T/C	0.763	-0.229	0.512	0.655	-1.109	0.666	0.096	1.74×10^{-4}	8.89×10^{-5}	0.051
Imboden et al (2012)	rs12615721	FEV1	Asthmatic	7.65×10^{-6}	No	G/A	0.926	-0.179	0.848	0.832	-0.227	1.106	0.838	-1.08×10^{-4}	1.46×10^{-4}	0.460
Imboden et al (2012)	rs10516809	FEV1	Asthmatic	8.67×10^{-6}	No	A/G	0.910	-0.941	0.763	0.217	-0.269	0.995	0.787	-1.54×10^{-4}	1.32×10^{-4}	0.245
Imboden et al (2012)	rs16856186	FEV1	Asthmatic	8.92×10^{-6}	No	T/G	0.921	0.586	0.843	0.487	0.455	1.096	0.678	-7.41×10^{-5}	1.47×10^{-4}	0.614
Imboden et al (2012)	rs158536	FEV1	Asthmatic	2.00×10^{-5}	No	T/C	0.643	0.842	0.454	0.064	1.352	0.591	**0.022 ****	1.53×10^{-5}	7.89×10^{-5}	0.847
Imboden et al (2012)	rs477725	FEV1	Asthmatic	3.00×10^{-5}	No	T/C	0.874	1.230	0.659	0.062	0.401	0.862	0.642	1.34×10^{-4}	1.14×10^{-4}	0.239
Imboden et al (2012)	rs9662589	FEV1	Asthmatic	5.00×10^{-5}	No	A/C	0.777	0.364	0.530	0.491	0.384	0.690	0.578	1.37×10^{-5}	9.19×10^{-5}	0.882
Imboden et al (2012)	rs777433	FEV1	Asthmatic	1.00×10^{-4}	No	T/C	0.648	0.467	0.459	0.309	0.523	0.597	0.381	6.60×10^{-5}	7.97×10^{-5}	0.407

Imboden et al (2012)	rs2658782	FEV1/FVC	General population	4.33×10^{-6}	No	G/T	0.793	-0.670	0.533	0.209	-0.733	0.695	0.292	2.89×10^{-5}	9.24×10^{-5}	0.755
Imboden et al (2012)	rs1867982	FEV1/FVC	General population	5.56×10^{-6}	No	G/A	0.891	-0.665	0.701	0.343	0.697	0.914	0.446	-2.69×10^{-4}	1.21×10^{-4}	**0.027 ** **
Imboden et al (2012)	rs12712969	FEV1/FVC	General population	7.08×10^{-6}	No	Not available in reference panel										
Imboden et al (2012)	rs10187654	FEV1/FVC	General population	8.87×10^{-6}	No	C/T	0.804	0.114	0.556	0.838	0.722	0.724	0.319	2.24×10^{-5}	9.64×10^{-5}	0.816
Imboden et al (2012)	rs356642	FEV1/FVC	General population	9.79×10^{-6}	No	G/A	0.815	0.430	0.549	0.433	0.135	0.716	0.850	7.88×10^{-5}	9.51×10^{-5}	0.408
Imboden et al (2012)	rs890515	FEV1/FVC	General population	1.00×10^{-5}	No	A/T	0.535	0.120	0.430	0.780	-0.570	0.561	0.309	1.14×10^{-4}	7.46×10^{-5}	0.125
Imboden et al (2012)	rs10738890	FEV1/FVC	General population	3.00×10^{-5}	No	T/C	0.621	-0.540	0.454	0.234	0.066	0.590	0.910	-1.47×10^{-4}	7.90×10^{-5}	0.063
Imboden et al (2012)	rs4831760	FEV1/FVC	Asthmatic	5.27×10^{-8}	No	T/C	0.694	0.715	0.482	0.138	0.220	0.627	0.726	1.38×10^{-4}	8.41×10^{-5}	0.101
Imboden et al (2012)	rs7144584	FEV1/FVC	Asthmatic	5.62×10^{-7}	No	C/T	0.897	0.022	0.712	0.976	-1.114	0.926	0.229	1.27×10^{-4}	1.24×10^{-4}	0.307
Imboden et al (2012)	rs1148186	FEV1/FVC	Asthmatic	7.28×10^{-6}	No	C/G	0.832	-1.335	0.590	**0.024 ** **	-1.134	0.769	0.140	-8.90×10^{-5}	1.02×10^{-4}	0.385

* Met genome-wide significance threshold in original paper

** Nominally significant (p<0.05) in BHS but not significant after Bonferroni correction

FEV_1, forced expiratory volume in one second. FVC, forced vital capacity. FEV_1/FVC, ratio of forced expiratory volume in one second to forced vital capacity. Beta, per-allele change in FEV_1 or FEV_1/FVC. SE, standard error.

Table S6b. Lookup results in BHS for previously reported regions [ever-smokers only] [2-4]

Paper	Top SNP in region in original paper	Associated trait (in original paper)	Cohort (in original paper)	P (in original paper)	Replicated (in original paper)	Coding/ non-coding allele	Freq	FEV1 (ml/yr)			FVC (ml/yr)			FEV1/FVC		
								Beta	SE	P	Beta	SE	P	Beta	SE	P
Hansel et al (2013)	rs7911302	FEV1 (% predicted)	COPD	**4.4×10^{-8} ***	No	C/A	0.578	1.104	0.681	0.105	0.403	0.875	0.645	2.41×10^{-4}	1.17×10^{-4}	0.039
Hansel et al (2013)	rs177852	FEV1 (% predicted)	COPD	**6.3×10^{-8} ***	No	C/T	0.664	0.140	0.734	0.849	0.870	0.940	0.355	-4.56×10^{-5}	1.26×10^{-4}	0.718
Hansel et al (2013)	rs2030436	FEV1 (% predicted)	COPD	3.2×10^{-7}	No	A/G	0.868	0.803	0.988	0.416	0.543	1.273	0.669	1.07×10^{-5}	1.69×10^{-4}	0.949
Hansel et al (2013)	rs12194741	FEV1 (% predicted)	COPD	1.8×10^{-6}	No	T/C	0.789	0.739	0.824	0.369	0.501	1.057	0.636	4.89×10^{-5}	1.41×10^{-4}	0.730
Hansel et al (2013)	rs2275456	FEV1 (% predicted)	COPD	3.1×10^{-6}	No	T/C	0.954	0.216	1.562	0.890	-1.302	1.994	0.514	3.47×10^{-4}	2.70×10^{-4}	0.200
Hansel et al (2013)	rs7331728	FEV1 (% predicted)	COPD	3.6×10^{-6}	No	A/G	0.832	1.177	0.891	0.186	1.739	1.144	0.128	-4.48×10^{-5}	1.53×10^{-4}	0.770
Hansel et al (2013)	rs4330400	FEV1 (% predicted)	COPD	7.3×10^{-6}	No	G/A	0.376	0.187	0.680	0.783	0.492	0.876	0.574	-2.01×10^{-5}	1.16×10^{-4}	0.863
Liao et al (2014)	rs1450439	FEV1	General population	4.38×10^{-6}	Not attempted	A/G	0.778	1.162	0.840	0.167	0.886	1.078	0.411	1.40×10^{-4}	1.44×10^{-4}	0.331
Liao et al (2014)	rs33794	FEV1	General population	6.07×10^{-6}	Not attempted	A/G	0.538	0.273	0.679	0.687	0.586	0.870	0.501	-1.66×10^{-4}	1.17×10^{-4}	0.156
Liao et al (2014)	rs10459348	FEV1	General population	9.84×10^{-6}	Not attempted	C/T	0.715	1.039	0.735	0.157	1.305	0.945	0.167	-2.70×10^{-5}	1.26×10^{-4}	0.831
Liao et al (2014)	rs1349345	FEV1	General population	1.39×10^{-5}	Not attempted	C/G	0.645	0.788	0.731	0.281	1.265	0.939	0.178	-2.40×10^{-5}	1.25×10^{-4}	0.848
Liao et al (2014)	rs7227539	FEV1	General population	1.58×10^{-5}	Not attempted	A/G	0.932	1.134	1.280	0.376	-0.048	1.651	0.977	2.58×10^{-4}	2.18×10^{-4}	0.237
Liao et al (2014)	rs9365009	FEV1	General population	1.98×10^{-5}	Not attempted	C/A	0.254	-0.728	0.767	0.343	0.197	0.986	0.842	-1.58×10^{-4}	1.31×10^{-4}	0.230
Liao et al (2014)	rs10779128	FEV1	General population	2.28×10^{-5}	Not attempted	A/G	0.775	1.279	0.782	0.102	1.881	1.008	0.062	3.76×10^{-5}	1.34×10^{-4}	0.779
Liao et al (2014)	rs17417768	FEV1/FVC	General population	1.15×10^{-5}	Not attempted	T/C	0.911	-0.135	1.136	0.905	2.049	1.460	0.160	-3.47×10^{-4}	1.95×10^{-4}	0.075
Liao et al (2014)	rs7117082	FEV1/FVC	General population	1.37×10^{-5}	Not attempted	G/T	0.917	0.800	1.225	0.514	1.185	1.575	0.452	1.13×10^{-4}	2.11×10^{-4}	0.591
Liao et al (2014)	rs11222969	FEV1/FVC	General population	1.66×10^{-5}	Not attempted	C/T	0.860	0.629	0.981	0.522	0.120	1.253	0.924	1.03×10^{-4}	1.70×10^{-4}	0.545
Liao et al (2014)	rs3760905	FEV1/FVC	General population	1.91×10^{-5}	Not attempted	G/T	0.829	0.812	1.031	0.431	0.034	1.326	0.980	2.77×10^{-4}	1.77×10^{-4}	0.116
Liao et al (2014)	rs7167951	FEV1/FVC	General population	2.30×10^{-5}	Not attempted	T/G	0.708	0.403	0.758	0.595	0.020	0.974	0.984	9.58×10^{-5}	1.30×10^{-4}	0.461

Liao et al (2014)	rs7945975	FEV1/FVC	General population	2.45x10^{-5}	Not attempted	A/G	0.860	-2.739	1.015	**0.007** **	-3.868	1.301	**0.003** **	-5.78x10^{-5}	1.75x10^{-4}	0.742
Liao et al (2014)	rs7620133	FEV1/FVC	General population	2.49x10^{-5}	Not attempted	C/T	0.685	0.538	0.717	0.453	0.885	0.920	0.336	2.41x10^{-5}	1.23x10^{-4}	0.845
Imboden et al (2012)	rs1889321	FEV1	General population	6.95x10^{-7}	No	T/C	0.738	0.198	0.734	0.787	0.731	0.943	0.438	-6.41x10^{-5}	1.26x10^{-4}	0.610
Imboden et al (2012)	rs9316500	FEV1	General population	4.81x10^{-6}	**Yes**	T/G	0.691	-1.074	0.720	0.136	-1.293	0.923	0.161	-8.41x10^{-5}	1.24x10^{-4}	0.498
Imboden et al (2012)	rs6785065	FEV1	General population	1.00x10^{-5}	No	C/T	0.770	0.282	0.775	0.716	0.911	0.995	0.360	1.74x10^{-5}	1.33x10^{-4}	0.896
Imboden et al (2012)	rs278037	FEV1	General population	2.00x10^{-5}	No	C/T	0.845	-0.248	0.936	0.791	-0.915	1.200	0.446	1.26x10^{-4}	1.60x10^{-4}	0.432
Imboden et al (2012)	rs7641198	FEV1	General population	3.00x10^{-5}	No	C/A	0.876	-0.947	1.021	0.354	0.259	1.314	0.844	-3.05x10^{-4}	1.75x10^{-4}	0.081
Imboden et al (2012)	rs421847	FEV1	General population	3.00x10^{-5}	No	G/A	0.725	0.881	0.762	0.248	-0.671	0.980	0.493	1.80x10^{-4}	1.31x10^{-4}	0.169
Imboden et al (2012)	rs496809	FEV1	General population	4.00x10^{-5}	No	C/T	0.943	-1.175	1.454	0.419	-2.138	1.862	0.251	1.85x10^{-4}	2.51x10^{-4}	0.460
Imboden et al (2012)	rs10933964	FEV1	General population	6.00x10^{-5}	No	A/G	0.499	-1.383	0.719	0.055	-1.328	0.926	0.152	-1.45x10^{-4}	1.23x10^{-4}	0.239
Imboden et al (2012)	rs10808265	FEV1	Asthmatic	1.66x10^{-6}	No	G/A	0.548	0.215	0.675	0.750	-0.364	0.867	0.674	1.94x10^{-4}	1.16x10^{-4}	0.093
Imboden et al (2012)	rs1902618	FEV1	Asthmatic	1.72x10^{-6}	No	A/G	0.810	-0.172	0.862	0.842	-0.136	1.107	0.902	-1.23x10^{-5}	1.48x10^{-4}	0.934
Imboden et al (2012)	rs3843306	FEV1	Asthmatic	5.11x10^{-6}	No	T/A	0.563	0.469	0.683	0.492	-0.790	0.876	0.367	1.07x10^{-4}	1.18x10^{-4}	0.361
Imboden et al (2012)	rs7006290	FEV1	Asthmatic	5.19x10^{-6}	No	G/A	0.683	0.790	0.723	0.274	0.978	0.930	0.293	1.24x10^{-4}	1.24x10^{-4}	0.316
Imboden et al (2012)	rs12436689	FEV1	Asthmatic	6.87x10^{-6}	No	T/C	0.763	-0.290	0.787	0.712	-0.821	1.011	0.417	1.42x10^{-4}	1.35x10^{-4}	0.294
Imboden et al (2012)	rs12615721	FEV1	Asthmatic	7.65x10^{-6}	No	G/A	0.926	-0.020	1.253	0.987	-1.228	1.617	0.447	9.51x10^{-5}	2.13x10^{-4}	0.655
Imboden et al (2012)	rs10516809	FEV1	Asthmatic	8.67x10^{-6}	No	A/G	0.910	-1.818	1.160	0.117	-0.440	1.495	0.769	-3.54x10^{-4}	1.99x10^{-4}	0.075
Imboden et al (2012)	rs16856186	FEV1	Asthmatic	8.92x10^{-6}	No	T/G	0.921	2.034	1.296	0.116	1.155	1.661	0.487	1.58x10^{-4}	2.23x10^{-4}	0.478
Imboden et al (2012)	rs158536	FEV1	Asthmatic	2.00x10^{-5}	No	T/C	0.643	0.840	0.694	0.226	1.724	0.892	0.053	-8.29x10^{-6}	1.19x10^{-4}	0.945
Imboden et al (2012)	rs477725	FEV1	Asthmatic	3.00x10^{-5}	No	T/C	0.874	1.249	1.008	0.215	-0.669	1.300	0.607	2.79x10^{-4}	1.72x10^{-4}	0.104
Imboden et al (2012)	rs9662589	FEV1	Asthmatic	5.00x10^{-5}	No	A/C	0.777	0.031	0.824	0.970	0.341	1.062	0.748	-1.50x10^{-4}	1.41x10^{-4}	0.285

Imboden et al (2012)	rs777433	FEV1	Asthmatic	1.00x10^{-4}	No	T/C	0.648	0.162	0.693	0.815	0.819	0.891	0.358	-1.37x10^{-5}	1.19x10^{-4}	0.908
Imboden et al (2012)	rs2658782	FEV1/FVC	General population	4.33x10^{-6}	No	G/T	0.793	-0.369	0.801	0.645	-0.450	1.030	0.662	5.91x10^{-5}	1.37x10^{-4}	0.667
Imboden et al (2012)	rs1867982	FEV1/FVC	General population	5.56x10^{-6}	No	G/A	0.891	-0.965	1.043	0.355	0.616	1.341	0.646	-3.10x10^{-4}	1.79x10^{-4}	0.083
Imboden et al (2012)	rs12712969	FEV1/FVC	General population	7.08x10^{-6}	No	Not available in reference panel										
Imboden et al (2012)	rs10187654	FEV1/FVC	General population	8.87x10^{-6}	No	C/T	0.804	0.650	0.853	0.446	1.703	1.097	0.121	5.33x10^{-5}	1.46x10^{-4}	0.716
Imboden et al (2012)	rs356642	FEV1/FVC	General population	9.79x10^{-6}	No	G/A	0.815	-0.114	0.831	0.891	-0.944	1.067	0.376	2.03x10^{-4}	1.42x10^{-4}	0.153
Imboden et al (2012)	rs890515	FEV1/FVC	General population	1.00x10^{-5}	No	A/T	0.535	1.446	0.662	**0.029 ** **	0.828	0.854	0.332	2.10x10^{-4}	1.13x10^{-4}	0.064
Imboden et al (2012)	rs10738890	FEV1/FVC	General population	3.00x10^{-5}	No	T/C	0.621	-0.956	0.691	0.166	-1.070	0.887	0.228	-1.02x10^{-4}	1.19x10^{-4}	0.393
Imboden et al (2012)	rs4831760	FEV1/FVC	Asthmatic	5.27x10^{-8}	No	T/C	0.694	0.908	0.749	0.225	0.692	0.961	0.472	7.77x10^{-5}	1.29x10^{-4}	0.546
Imboden et al (2012)	rs7144584	FEV1/FVC	Asthmatic	5.62x10^{-7}	No	C/T	0.897	-0.874	1.058	0.409	-1.822	1.358	0.180	-4.25x10^{-5}	1.82x10^{-4}	0.815
Imboden et al (2012)	rs1148186	FEV1/FVC	Asthmatic	7.28x10^{-6}	No	C/G	0.832	-1.717	0.920	0.062	-1.688	1.181	0.153	-7.94x10^{-5}	1.58x10^{-4}	0.616

* Met genome-wide significance threshold in original paper

** Nominally significant ($p<0.05$) in BHS but not significant after Bonferroni correction

FEV$_1$, forced expiratory volume in one second. FVC, forced vital capacity. FEV$_1$/FVC, ratio of forced expiratory volume in one second to forced vital capacity. Beta, per-allele change in FEV$_1$ or FEV$_1$/FVC. SE, standard error.

Table S6c. Lookup results in BHS for previously reported regions [never-smokers only] [2-4]

Paper	Top SNP in region in original paper	Trait associated with (in original paper)	Cohort (in original paper)	P (in original paper)	Replicated (in original paper)	Coding/ non-coding allele	Freq	FEV1 (ml/yr)			FVC (ml/yr)			FEV1/FVC		
								Beta	SE	P	Beta	SE	P	Beta	SE	P
Hansel et al (2013)	rs7911302	FEV1 (% predicted)	COPD	**$4.4x10^{-8}$ ***	No	C/A	0.578	0.262	0.551	0.634	0.556	0.732	0.448	$-4.33x10^{-5}$	$1.01x10^{-4}$	0.669
Hansel et al (2013)	rs177852	FEV1 (% predicted)	COPD	**$6.3x10^{-8}$ ***	No	C/T	0.664	0.893	0.587	0.128	0.827	0.779	0.288	$9.99x10^{-5}$	$1.08x10^{-4}$	0.355
Hansel et al (2013)	rs2030436	FEV1 (% predicted)	COPD	$3.2x10^{-7}$	No	A/G	0.868	-1.193	0.813	0.143	-0.140	1.081	0.897	$-2.80x10^{-4}$	$1.49x10^{-4}$	0.060
Hansel et al (2013)	rs12194741	FEV1 (% predicted)	COPD	$1.8x10^{-6}$	No	T/C	0.789	-0.221	0.655	0.736	-0.687	0.872	0.431	$1.30x10^{-4}$	$1.20x10^{-4}$	0.278
Hansel et al (2013)	rs2275456	FEV1 (% predicted)	COPD	$3.1x10^{-6}$	No	T/C	0.954	-0.188	1.325	0.887	-2.162	1.760	0.219	$4.20x10^{-4}$	$2.44x10^{-4}$	0.085
Hansel et al (2013)	rs7331728	FEV1 (% predicted)	COPD	$3.6x10^{-6}$	No	A/G	0.832	-1.071	0.757	0.157	-1.051	1.006	0.296	$-1.53x10^{-4}$	$1.39x10^{-4}$	0.270
Hansel et al (2013)	rs4330400	FEV1 (% predicted)	COPD	$7.3x10^{-6}$	No	G/A	0.376	-0.052	0.576	0.928	-0.457	0.766	0.551	$2.30x10^{-5}$	$1.05x10^{-4}$	0.827
Liao et al (2014)	rs1450439	FEV1	General population	$4.38x10^{-6}$	Not attempted	A/G	0.778	0.598	0.659	0.365	-0.339	0.877	0.699	$2.58x10^{-4}$	$1.21x10^{-4}$	**0.033****
Liao et al (2014)	rs33794	FEV1	General population	$6.07x10^{-6}$	Not attempted	A/G	0.538	0.663	0.561	0.237	0.681	0.746	0.361	$9.38x10^{-5}$	$1.03x10^{-4}$	0.363
Liao et al (2014)	rs10459348	FEV1	General population	$9.84x10^{-6}$	Not attempted	C/T	0.715	0.133	0.601	0.825	-0.336	0.800	0.675	$1.60x10^{-4}$	$1.10x10^{-4}$	0.145
Liao et al (2014)	rs1349345	FEV1	General population	$1.39x10^{-5}$	Not attempted	C/G	0.645	0.714	0.585	0.222	0.367	0.779	0.638	$1.39x10^{-4}$	$1.07x10^{-4}$	0.194
Liao et al (2014)	rs7227539	FEV1	General population	$1.58x10^{-5}$	Not attempted	A/G	0.932	0.774	1.056	0.463	-1.600	1.402	0.254	$6.37x10^{-4}$	$1.95x10^{-4}$	**0.001****
Liao et al (2014)	rs9365009	FEV1	General population	$1.98x10^{-5}$	Not attempted	C/A	0.254	-0.413	0.637	0.516	0.418	0.847	0.622	$-1.50x10^{-4}$	$1.17x10^{-4}$	0.198
Liao et al (2014)	rs10779128	FEV1	General population	$2.28x10^{-5}$	Not attempted	A/G	0.775	1.092	0.675	0.106	1.376	0.897	0.125	$-6.22x10^{-5}$	$1.23x10^{-4}$	0.614
Liao et al (2014)	rs17417768	FEV1/FVC	General population	$1.15x10^{-5}$	Not attempted	T/C	0.911	0.935	1.001	0.350	1.267	1.329	0.341	$1.52x10^{-4}$	$1.84x10^{-4}$	0.410
Liao et al (2014)	rs7117082	FEV1/FVC	General population	$1.37x10^{-5}$	Not attempted	G/T	0.917	-0.611	1.027	0.552	-1.650	1.367	0.228	$1.57x10^{-4}$	$1.88x10^{-4}$	0.403
Liao et al (2014)	rs11222969	FEV1/FVC	General population	$1.66x10^{-5}$	Not attempted	C/T	0.860	-0.556	0.816	0.496	-0.403	1.088	0.711	$7.19x10^{-5}$	$1.49x10^{-4}$	0.629
Liao et al (2014)	rs3760905	FEV1/FVC	General population	$1.91x10^{-5}$	Not attempted	G/T	0.829	-1.083	0.825	0.189	-1.636	1.104	0.138	$4.63x10^{-6}$	$1.50x10^{-4}$	0.975
Liao et al (2014)	rs7167951	FEV1/FVC	General population	$2.30x10^{-5}$	Not attempted	T/G	0.708	-0.337	0.602	0.575	-0.394	0.800	0.623	$-3.43x10^{-5}$	$1.10x10^{-4}$	0.756

Liao et al (2014)	rs7945975	FEV1/FVC	General population	2.45x10^{-5}	Not attempted	A/G	0.860	1.139	0.780	0.144	1.642	1.037	0.113	6.83x10^{-5}	1.43x10^{-4}	0.633
Liao et al (2014)	rs7620133	FEV1/FVC	General population	2.49x10^{-5}	Not attempted	C/T	0.685	-0.127	0.590	0.829	0.231	0.785	0.769	-1.21x10^{-4}	1.08x10^{-4}	0.262
Imboden et al (2012)	rs1889321	FEV1	General population	6.95x10^{-7}	No	T/C	0.738	0.205	0.638	0.748	1.647	0.849	0.052	-2.46x10^{-4}	1.16x10^{-4}	**0.034****
Imboden et al (2012)	rs9316500	FEV1	General population	4.81x10^{-6}	**Yes**	T/G	0.691	0.460	0.597	0.440	0.470	0.794	0.554	9.27x10^{-5}	1.09x10^{-4}	0.396
Imboden et al (2012)	rs6785065	FEV1	General population	1.00x10^{-5}	No	C/T	0.770	-0.963	0.661	0.145	-1.085	0.877	0.216	-2.49x10^{-5}	1.22x10^{-4}	0.838
Imboden et al (2012)	rs278037	FEV1	General population	2.00x10^{-5}	No	C/T	0.845	-0.352	0.745	0.636	0.266	0.993	0.789	-1.76x10^{-4}	1.35x10^{-4}	0.193
Imboden et al (2012)	rs7641198	FEV1	General population	3.00x10^{-5}	No	C/A	0.876	0.136	0.836	0.870	0.798	1.112	0.473	-1.48x10^{-4}	1.53x10^{-4}	0.332
Imboden et al (2012)	rs421847	FEV1	General population	3.00x10^{-5}	No	G/A	0.725	-0.009	0.622	0.988	-0.286	0.828	0.730	-2.85x10^{-5}	1.14x10^{-4}	0.802
Imboden et al (2012)	rs496809	FEV1	General population	4.00x10^{-5}	No	C/T	0.943	-1.457	1.146	0.203	-0.656	1.518	0.666	-2.18x10^{-4}	2.12x10^{-4}	0.304
Imboden et al (2012)	rs10933964	FEV1	General population	6.00x10^{-5}	No	A/G	0.499	-0.076	0.574	0.895	-0.547	0.763	0.474	1.23x10^{-4}	1.05x10^{-4}	0.244
Imboden et al (2012)	rs10808265	FEV1	Asthmatic	1.66x10^{-6}	No	G/A	0.548	-0.944	0.561	0.092	-0.837	0.745	0.262	-9.23x10^{-5}	1.03x10^{-4}	0.371
Imboden et al (2012)	rs1902618	FEV1	Asthmatic	1.72x10^{-6}	No	A/G	0.810	-0.016	0.696	0.981	0.167	0.927	0.857	-1.69x10^{-5}	1.27x10^{-4}	0.894
Imboden et al (2012)	rs3843306	FEV1	Asthmatic	5.11x10^{-6}	No	T/A	0.563	-0.186	0.560	0.740	0.156	0.745	0.834	-1.02x10^{-4}	1.02x10^{-4}	0.316
Imboden et al (2012)	rs7006290	FEV1	Asthmatic	5.19x10^{-6}	No	G/A	0.683	0.251	0.601	0.676	0.834	0.799	0.296	-4.82x10^{-5}	1.10x10^{-4}	0.661
Imboden et al (2012)	rs12436689	FEV1	Asthmatic	6.87x10^{-6}	No	T/C	0.763	0.218	0.643	0.735	-0.925	0.854	0.279	2.17x10^{-4}	1.18x10^{-4}	0.065
Imboden et al (2012)	rs12615721	FEV1	Asthmatic	7.65x10^{-6}	No	G/A	0.926	-0.486	1.108	0.661	0.611	1.475	0.679	-3.66x10^{-4}	2.02x10^{-4}	0.071
Imboden et al (2012)	rs10516809	FEV1	Asthmatic	8.67x10^{-6}	No	A/G	0.910	-0.371	0.964	0.701	-0.288	1.282	0.822	-3.26x10^{-5}	1.77x10^{-4}	0.854
Imboden et al (2012)	rs16856186	FEV1	Asthmatic	8.92x10^{-6}	No	T/G	0.921	-0.669	1.057	0.527	-0.121	1.404	0.931	-2.90x10^{-4}	1.95x10^{-4}	0.136
Imboden et al (2012)	rs158536	FEV1	Asthmatic	2.00x10^{-5}	No	T/C	0.643	0.691	0.571	0.226	0.877	0.759	0.248	1.40x10^{-5}	1.05x10^{-4}	0.894
Imboden et al (2012)	rs477725	FEV1	Asthmatic	3.00x10^{-5}	No	T/C	0.874	0.994	0.829	0.230	1.018	1.108	0.358	6.68x10^{-7}	1.51x10^{-4}	0.996
Imboden et al (2012)	rs9662589	FEV1	Asthmatic	5.00x10^{-5}	No	A/C	0.777	0.802	0.656	0.221	0.684	0.871	0.433	1.50x10^{-4}	1.21x10^{-4}	0.214
Imboden et al (2012)	rs777433	FEV1	Asthmatic	1.00x10^{-4}	No	T/C	0.648	0.461	0.586	0.431	-0.127	0.778	0.871	1.19x10^{-4}	1.07x10^{-4}	0.268

Imboden et al (2012)	rs2658782	FEV1/FVC	General population	4.33x10^{-6}	No	G/T	0.793	-1.010	0.682	0.139	-1.277	0.908	0.160	1.79x10^{-5}	1.25x10^{-4}	0.886
Imboden et al (2012)	rs1867982	FEV1/FVC	General population	5.56x10^{-6}	No	G/A	0.891	-0.673	0.910	0.460	0.318	1.213	0.793	-2.21x10^{-4}	1.66x10^{-4}	0.183
Imboden et al (2012)	rs12712969	FEV1/FVC	General population	7.08x10^{-6}	No	Not available in reference panel										
Imboden et al (2012)	rs10187654	FEV1/FVC	General population	8.87x10^{-6}	No	C/T	0.804	-0.371	0.695	0.594	-0.208	0.926	0.822	-2.87x10^{-5}	1.27x10^{-4}	0.821
Imboden et al (2012)	rs356642	FEV1/FVC	General population	9.79x10^{-6}	No	G/A	0.815	0.955	0.698	0.171	1.401	0.929	0.131	-7.15x10^{-5}	1.28x10^{-4}	0.575
Imboden et al (2012)	rs890515	FEV1/FVC	General population	1.00x10^{-5}	No	A/T	0.535	-1.211	0.537	**0.024 ****	-1.814	0.714	**0.011 ****	-9.31x10^{-6}	9.86x10^{-5}	0.925
Imboden et al (2012)	rs10738890	FEV1/FVC	General population	3.00x10^{-5}	No	T/C	0.621	0.058	0.574	0.920	1.266	0.762	0.096	-1.48x10^{-4}	1.06x10^{-4}	0.161
Imboden et al (2012)	rs4831760	FEV1/FVC	Asthmatic	5.27x10^{-8}	No	T/C	0.694	0.794	0.600	0.186	-0.056	0.796	0.944	2.27x10^{-4}	1.10x10^{-4}	**0.040****
Imboden et al (2012)	rs7144584	FEV1/FVC	Asthmatic	5.62x10^{-7}	No	C/T	0.897	0.093	0.927	0.920	-1.461	1.227	0.234	2.93x10^{-4}	1.71x10^{-4}	0.087
Imboden et al (2012)	rs1148186	FEV1/FVC	Asthmatic	7.28x10^{-6}	No	C/G	0.832	-0.734	0.730	0.315	-0.487	0.975	0.618	-7.25x10^{-5}	1.33x10^{-4}	0.587

* Met genome-wide significance threshold in original paper

** Nominally significant ($p<0.05$) in BHS but not significant after Bonferroni correction

FEV_1, forced expiratory volume in one second. FVC, forced vital capacity. FEV_1/FVC, ratio of forced expiratory volume in one second to forced vital capacity. Beta, per-allele change in FEV_1 or FEV_1/FVC. SE, standard error.

<u>References</u>

1. Soler Artigas M, Loth DW, Wain LV, et al. Genome-wide association and large-scale follow up identifies 16 new loci influencing lung function. *Nat Genet* 2011;**43**(11):1082-90.
2. Hansel NN, Ruczinski I, Rafaels N, et al. Genome-wide study identifies two loci associated with lung function decline in mild to moderate COPD. *Hum Genet* 2013;**132**(1):79-90.
3. Liao SY, Lin X, Christiani DC. Genome-wide association and network analysis of lung function in the Framingham Heart Study. *Genet Epidemiol* 2014;**38**(6):572-8.
4. Imboden M, Bouzigon E, Curjuric I, et al. Genome-wide association study of lung function decline in adults with and without asthma. *J Allergy Clin Immunol* 2012;**129**(5):1218-28.
